# Transcriptional Regulation of Notch1 Expression by Nkx6.1 in Neural Stem/Progenitor Cells during Ventral Spinal Cord Development

**DOI:** 10.1038/srep38665

**Published:** 2016-12-07

**Authors:** Ying Li, Evangeline Tzatzalos, Kelvin Y. Kwan, Martin Grumet, Li Cai

**Affiliations:** 1 Department of Biomedical Engineering, Rutgers University, 599 Taylor Road, Piscataway, NJ 08854, USA; 2 W.M. Keck Center for Collaborative Neuroscience, Department of Cell Biology and Neuroscience, Rutgers University, 604 Allison Road, Piscataway, NJ 08854, USA.

## Abstract

Notch1 signaling plays a critical role in maintaining and determining neural stem/progenitor cell (NSPC) fate, yet the transcriptional mechanism controlling Notch1 specific expression in NSPCs remains incomplete. Here, we show transcription factor Nkx6.1 interacts with a cis-element (CR2, an evolutionarily conserved non-coding fragment in the second intron of Notch1 locus) and regulates the expression of Notch1 in ventral NSPCs of the developing spinal cord. We show that the Notch1 expression is modulated by the interaction of Nkx6.1 with a 139 bp enhancer sequence within CR2. Knockdown or overexpression of Nkx6.1 leads to down- or up-regulated Notch1 expression, respectively. In CR2-GFP transgenic mouse, GFP expression was found prominent in the ventricular zone and neural progenitor cells from embryonic day 9.5 to postnatal day 7. GFP+ cells were mainly neural progenitors for interneurons and not for motoneurons or glial cells. Moreover, GFP expression persisted in a subset of ependymal cells in the adult spinal cord, suggesting that CR2 is active in both embryonic and adult NSPCs. Together our data reveal a novel mechanism of Notch1 transcriptional regulation in the ventral spinal cord by Nkx6.1 via its binding with Notch1 enhancer CR2 during embryonic development.

Notch1 is a member of the Notch protein family which encodes a single-pass trans-membrane receptor. Notch1 signaling plays a critical role in the development of the central nervous system (CNS) by inhibiting neuronal progenitor differentiation, maintaining radial glia identity, specifying glial cell type, promoting apoptotic cell death and regulating axonal guidance of post-mitotic neurons^1^,^2^,^3^,^4^,^5^,^6^,^7^. In the spinal cord, in additional to its role in neural stem cells, Notch1 is involved in fate determination of dorsal interneurons and V2b interneurons^8^,^9^,^10^. Notch1 deficiency results in a premature neuronal differentiation in the ventral spinal cord and a gradual depletion of the ventral central canal^5^. However, despite the importance of Notch1 pathway, transcriptional regulation of Notch1 expression is not completely understood. Usually, transcription factors function by binding to gene regulatory DNA elements, e.g., promoters, enhancers. Often these *cis*-elements are evolutionarily conserved^11^,^12^. We have previously identified an evolutionarily conserved *cis*-element in the second intron of Notch1 gene (Notch1CR2, or CR2, a 399 bp non-coding DNA fragment)^13^. CR2 regulates gene expression in neural stem/progenitor cells (NSPCs) and was predominantly active in the GABAergic interneuron progenitors in the ganglionic eminence during neocortical development^13^.

In the spinal cord, differentiation of neurons and glial cells are regulated by a transcription factor network including Notch1, Nkx6.1, Pax6, Foxn4 and Dll4, etc.^14^. Nkx6.1 in particular, is critical for the fate determination of progenitor cells in ventral interneuron layer 2 and 3 (V2, V3)^15^. Nkx6.1 deletion leads to the loss of V2 and motoneuron (MN) markers such as Lhx3, Isl1/2 and Hb9^15^, while over-expression of Nkx6.1 leads to an increase of V2, MN markers and decrease of V1 marker^16^.

In this study, we investigated the Notch1 transcription regulation by CR2 in the developing mouse and chick spinal cord. We show that a 139 bp fragment within CR2 contains binding sites for transcription factor Nkx6.1. The interaction of this fragment with Nkx6.1 is essential for CR2 gene regulatory activity in regulating Notch1 in the NSPCs which develop into interneurons. Our study provides a novel mechanism of Notch1 gene regulation in NSPCs by Nkx6.1-CR2 interaction during the development of the spinal cord.

## Material and Methods

### Mouse strains and tissue preparation

All experimental protocols were approved by the Institutional Animal Care and Use Committee (IACUC) and the Institutional Biosafety Committee at Rutgers University. All animal work was conducted in accordance with relevant guidelines and regulations of the IACUC. The CR2-GFP transgenic mouse (*Mus musculus*) strain was generated as previously described^13^ and maintained in our lab. Embryonic and neonatal spinal cords were obtained from the transgenic animals via microsurgical dissection. They were washed in 1X PBS and fixed with 4% (w/v) paraformaldehyde overnight. Fixed tissues were washed again and then cryopreserved in 30% (w/v) sucrose overnight. Afterwards, the spinal cord tissue was embedded in cryo-preserving media (Tissue Tek® OCT compound) and kept frozen at −80 °C.

### Immunohistochemistry

Frozen spinal cord tissue was sectioned transversely (10–12 μm in thickness) using a cryostat (ThermoScientific) and air dried. Sections were blocked and permeablized for 1 hour (hr) in blocking buffer containing 10% donkey serum, 0.1% TritonX-100, and 0.1% Tween 20 at room temperature. Afterwards, sections were incubated with primary antibodies overnight at 4 °C. Following three 10-min washes in PBS, sections were incubated in the blocking buffer containing corresponding fluorophore-conjugated secondary antibodies for 1 hr at room temperature. Slides were then washed for three times with PBS (10-min each), and mounted with mounting media (Vector Laboratories) in the presence or absence of DAPI (to label the nuclei). The following primary antibodies were used: anti-Notch1 (rabbit, polyclonal, 1:100, 6014-R), anti-Gata2 (rabbit, polyclonal, 1:200, sc-9008x) and anti-Chx10 (goat, polyclonal, 1:300, sc-21692) from Santa Cruz Biotechnology, Inc.; anti-En1 (mouse, monoclonal, 1:1000, 4G11-c), anti-Evx1 (mouse, monoclonal, 1:10, 99.1–3A2), anti-Isl1 (mouse, monoclonal, 1:100, 39.4D5), anti-Nkx2.2 (mouse, monoclonal, 1:10, 74.5A5), anti-Nkx6.1 (mouse, monoclonal, 1:25, F55A12), anti-Pax7 (mouse, monoclonal, 1:25) and anti-Pax6 (mouse, monoclonal, 1:15) from Developmental Studies Hybridoma Bank (DSHB); anti-Brn3a (mouse, monoclonal, 1:100, MAB1585), anti-NeuN (mouse, monoclonal, 1:1000, MAB377) and anti-Sox2 (mouse, monoclonal, 1:500, MAB4343) from Millipore; anti-GFP (goat, polyclonal, 1:1000, AB5450) from Abcam; anti-GFP (rabbit, polyclonal, 1:500, a11122) from Invitrogen; anti-S100b (mouse, monoclonal, 1:1000, S2532) from Sigma; anti-Pax2 (rabbit, polyclonal, 1:250, 71–6000) from Zymed; anti-Olig2 (rabbit, polyclonal, 1:10000, gift from Dr. Charles Stiles at Harvard University). The staining with anti-Olig2 antibody requires pre-heating of slides with 1 mM Tris-EDTA buffer (PH 8.5) at 96 °C for 10-min to retrieve the antigen. Images were captured using a Zeiss Axio Imager M1 fluorescence microscope and visualized with AxioVision 4.8.

### Cell counting and statistical analysis

Cell counting was performed manually on T8~T10 spinal cord sections based on the DAPI-stained nuclei. For each cellular marker, 3~5 sections from at least 3 animals at each time point were counted. Since GFP protein is expressed in the cytoplasm while Notch1 staining is located on the cell surface, DAPI nuclei staining confirmed the double labeling of GFP and Notch1. Quantitative data were presented as mean ± standard deviation. Significance (p-value) was determined by Student’s t-test.

### Plasmid construction

Designed sub-regions (Table S1) and mutated CR2.a sequences (Table S2) were sub-cloned into an expression vector which contains a minimal β-globin promoter (βGP) and a GFP reporter gene. Clones were confirmed by PCR and sequencing. A transfection control construct with a constitutively active CAG promoter and a DsRed reporter was also generated.

### *In ovo* electroporation

SPF fertilized eggs were purchased (Sunrise Farms, Inc., New York) and incubated at 37 °C with 60% humidity. The developmental stages of the chicks were determined according to stages established by Hamilton and Hamburger^17^. In ovo electroporation was performed on E2 (HH11-12) or E5 (HH26-27) chick embryos following the protocol^18^ with modifications. Mixed DNA for CR2 sub-regions (Table S1) or mutated CR2.a sequences (Table S2) contains ~2.5 μg μl^−1^ experimental plasmid, ~0.2 μg μl^−1^ transfection control plasmid and 0.025% Fast Green dye. Mixed DNA for shRNA assay contains ~2.5 μg μl^−1^ experimental shRNA plasmid, ~2.5 μg μl^−1^ CR2.a-GFP plasmid and 0.025% Fast Green dye. Mixed DNA for overexpression assay contains ~2.5 μg μl^−1^ factor expressing plasmid, ~2.5 μg μl^−1^ CR2.a-GFP plasmid and 0.025% Fast Green dye. Injection of the mixed DNA was performed to the middle region of chick neural tube (region with somites), following by electroporation of five 12 V pulses. Eggs with E2 injection were harvested on E4 or E5. Eggs with E5 injection were harvested on E6. The chick embryos were examined under a fluorescent whole mount microscope (Leica, MZ16FA). The chick embryo tissues were then washed in 1x PBS and fixed with 4% (w/v) paraformaldehyde for 1 hr. Processes following fixation are the same as preparing mouse spinal cord tissue.

### Electrophoretic mobility shift assay (EMSA)

ESMA was performed with the designed double strand probes (Table S3) and nuclear extract from E15.5 mouse spinal cord. Single strand probes were first synthesized by IDT (Piscataway, NJ). They are biotinylated using the Biotin 3´ End DNA Labeling Kit (Thermo Fisher Scientific Inc, IL) and annealed at room temperature for one hour. Biotin-labeled double strand probes were stored at −20 °C for no longer than 1 week. Unlabeled single stranded probes were also annealed at room temperature for one hour and used as competitors. The ratio of labeled probes and unlabeled probes was 1: 20. EMSA is performed using the LightShift Chemiluminescent EMSA Kit (Thermo Fisher Scientific Inc, IL) following the manufactory’s instruction. Reaction mixtures were then loaded onto 8% non-denaturing polyacrylamide gel and run at 100 V for 120–150 min at 4 °C.

### RNAi-mediated gene knockdown

For RNA interference assays, two 23~29-mer shRNA hairpins were designed based on chick mRNA for each of the Nkx6.1and Phox2b genes (Table S4). Each of them was sub-cloned into a shRNA expressing vector (Origene TR30014) which contains a RFP reporter. Clones were confirmed by PCR and sequencing. A negative control construct with scrambled-shRNA (Origene TR30015) was used. Normal *in ovo* electroporation procedure described above is performed to transfect cells in chick neural tube. The two shRNA constructs designed for each transcription factor were used separately in the transfection.

### Nkx6.1 overexpression

A Nkx6.1 overexpression construct, Tet-O-FUW-Nkx6.1^19^, was obtained from Addgene (plasmid #45846) and injected into chick neural tube on various stages followed by *in ovo* electroporation as described above. DNA mixture contains ~2.5 μg μl^−1^ Tet-O-FUW-Nkx6.1, ~0.2 μg μl^−1^ CAG-DsRed and 0.025% fast green dye. Immunohistochemistry and qPCR analysis were used to confirm the successful overexpression of Nkx6.1.

### Quantitative reverse transcription PCR (qRT-PCR)

For qRT-PCR, total RNA was extracted from mouse spinal cord tissues at E15.5, P0 and P14 using Tri Reagent Solution (Ambion). First strand cDNA library was constructed by reverse transcription with qScript cDNA SuperMix (Quanta Biosciences) and used as the template. qPCR was performed on a Roche 480 LightCycler using SYBR Green FastMix (Applied Biosystems) following manufactory’s instruction using primers designed for GFP, Notch1, GAPDH and other genes (Table S5).

Similarly, qRT-PCR analysis was performed with chick spinal cord samples. E5 chick embryos were transfected with the shNkx6.1, scr-shRNA (with RFP reporter) or the Tet-O-FUW-Nkx6.1 construct (co-transfect with pCAG-DsRed) using *in ovo* electroporation technique. Electroporation results in RFP + cells in half of the spinal cord. On E6, the RFP+ half of the spinal cord tissues were harvested for total RNA extraction. cDNA library was constructed as described above and used as template to generate a 20-cycle PCR product for the following qPCR.

For all qPCR experiments, results were reported as relative threshold cycles (∆Ct). It is calculated by normalizing the threshold cycles based on the GAPDH expression. Each data point contains at least 3 samples with 3 replicates.

### Chromatin immunoprecipatation (ChIP)

Pregnant wild-type CD-1 mice (Charles River) were harvested and the spinal cord of twenty-eight E14.5 embryos were collected and pooled for later steps. The MAGnify Chromatin Immunoprecipitation system (Invitrogen) was used to extract the chromatin and precipitation. Experiment was performed following the manufacturer’s instructions with modifications. Spinal cord tissue was homogenized by pipetting. Dissociated cells were cross-linked by 1% paraformaldehyde at room temperature for 10 min and quenched by 0.125 M glycine. Cells were then incubated in Lysis Buffer containing protease inhibitor for 5 min on ice. Sonication was performed with Bioruptor UCD-200 (Diagenode) for three eight-minutes sonicating with cycles of 30 s ‘ON’ and 30 s ‘OFF’. This yielded 200–800 bp fragments of sheared chromatin. 6 μg of each antibody was used for immunoprecipitation (IP) including anti-Nkx6.1 (C-terminus), Nkx6.1 (N-terminus), H3K9Ac and ms IgG. IP was performed overnight at 4 °C. Final product DNA was purified with beads and reconstitute in 150 μl DNA Elution Buffer. Afterwards, DNA from each IP was amplified by qRT-PCR with triplicates using the CR2.a primers (Table S1).

## Results

### Functional core sequence of Notch1 enhancer CR2

To elucidate the mechanism underlying transcriptional regulation of Notch1 expression in NSPCs, we analyzed the activity of a Notch1 enhancer CR2 during spinal cord development. CR2 is a fragment of evolutionarily conserved non-coding DNA located in the second intron of Notch1 gene locus and its gene regulatory activity has been shown in the interneuron progenitors of the developing neocortex^13^. We first determined the minimum sequence and protein factors required for CR2 activity and analyzed protein binding and gene regulatory activities within sub-regions of CR2. Using MatInspector (Genomatix, Germany), we identified a total of 173 potential transcription factor binding sites (TFBSs) on the 399 bp CR2 (Table S6), which correspond to 126 unique transcription factors (TFs). Twenty of these 126 TFs are expressed in the developing spinal cord during mid-late embryonic stages (E10-E20) (Fig. S1). We thus focused on these 20 factors for further analysis. In particular, the binding sites of seven of these TFs are 100% conserved between mouse and chick (red font in Fig. S1), suggesting that these 7 factors may have critical function in the spinal cord^11^,^12^.

**Figure 1:**
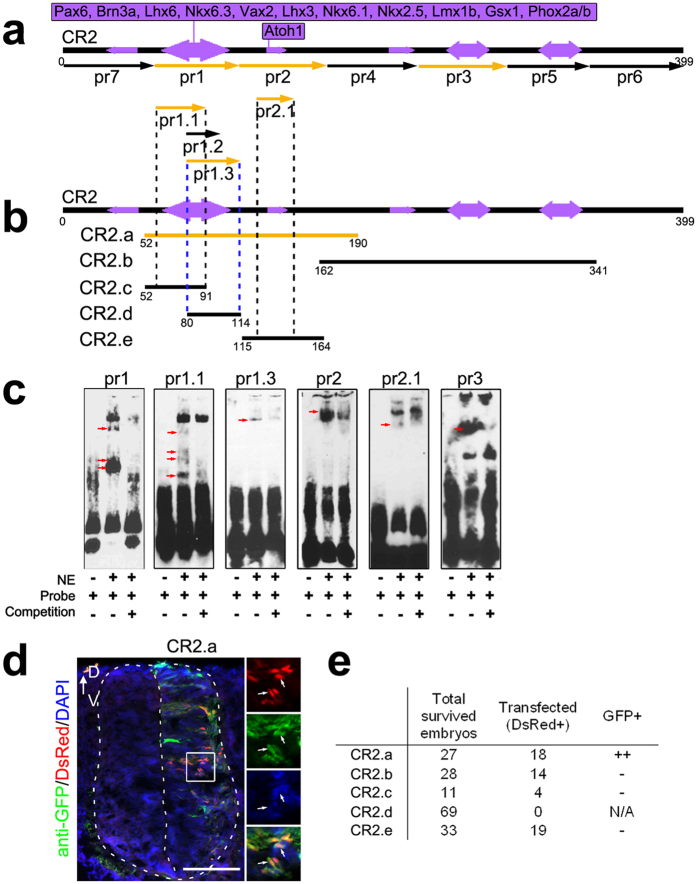
A 139 bp sequence in CR2 is required for its gene regulatory activity. Sub-regions of CR2 were tested individually by EMSA and reporter assay with *in ovo* electroporation for their role in gene regulation. Eleven EMSA probes ((**a**) pr1–7, pr1.1–1.3, pr2.1) and five CR2 sub-regions ((**b**) CR2.a-2.e) were designed based on the location of predicted transcription factor binding sites (purple arrows, see Fig. S1 for detail) on CR2 (black bar). CR2.c, CR2.d and CR2.e contains the sequences of pr1.1, pr1.3 and pr2.1, respectively (indicated by the dotted lines). Probes and sub-regions that showed positive results in EMSA or reporter assay are colored in yellow. Multiple specific binding bands (red arrows) were detected in EMSA using E15.5 mouse spinal cord nuclear extracts with pr1, pr1.1, pr1.3, pr2, pr2.1 and pr3 ((**c**) and Fig. S2a). A cross section of E5 chick spinal cord shows that only CR2.a-GFP construct resulted in GFP expression by reporter assay with *in ovo* electroporation at E2 ((**d**) and Fig. S2b). CAG-DsRed construct was used as a transfection control. Arrows indicate CR2.a-GFP+ cells. Dotted line outlines the neural tube of chick embryo. A table summarizes the results of all the tested sub-regions of CR2 (**e**). The transcription factors with binding sites in CR2.a region are listed on the top (**a**). NE, nuclear extract; D, dorsal; V, ventral. Scale bar = 50 μm.

We next performed electrophoretic mobility shift assays (EMSA) using nuclear protein extracts from E15.5 mouse spinal cord. A total of seven probes were designed based on the TFBS analysis (pr1–7, Fig. 1a and Table S3). Sequence specific binding activity was detected with pr1–3 (Fig. 1c, Fig. S2a). Since pr1 contains a cluster of 12 TFBSs (Fig. 1a, Fig. S1) and showed multiple bands in EMSA (Fig. 1c, it was further dissected into pr1.1, pr1.2 and pr1.3 (Fig. 1a). Protein binding activities were detected with pr1.1 and pr1.3 (Fig. 1a, Fig. S2a). Multiple bands were observed with pr1.1, suggesting that multiple proteins interact with pr1.1 at multiple locations. Thereafter, sub-regions of CR2 with positive binding in EMSA (CR2.a-e, Fig. 1b and Table S1) were selected for a GFP reporter assay to determine their transcriptional regulatory activity *in vivo*. The reporter plasmid DNA mixture containing one of the sub-regions (CR2.x-GFP) and a positive control (CAG-DsRed) was injected and electroporated to transfect the developing neural tube of E2 chick embryos. Results show that only CR2.a was able to drive GFP expression in E5 chick spinal cord (Fig. 1d,e). No GFP expression was detected in the samples transfected with constructs containing CR2.b or sub-regions of CR2.a (CR2.c, CR2.e) (Fig. S2b). In addition, no reporter expression (neither GFP nor DsRed) was observed in any of the chick embryos (n = 69) transfected with the CR2.d construct. It suggests that CR2.d may interfere with the proper gene expression since 55% of chick embryos transfected with other constructs showed the control DsRed expression (Fig. 1e). Thus, CR2.a was determined as the minimum required sequence for interaction with multiple TFBSs and is required for CR2 activation.

### Nkx6.1 regulates CR2.a activity

**Figure 2:**
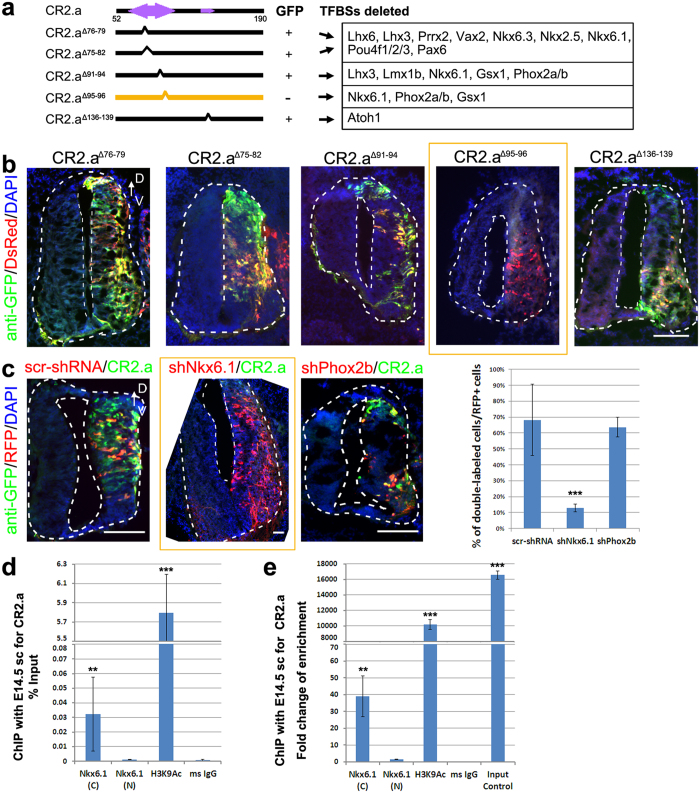
Nkx6.1 is required for CR2.a activity. Site-direct mutagenesis, shRNA gene knockdown and chromatin immunoprecipitation (ChIP) were performed in chick developing spinal cord to determine the functional role of the predicted factor binding sites and their interacting factors. 2~8 bp in the core region of factor binding sites on CR2.a were deleted (**a**) to generate mutated reporter constructs with human beta-globin basic promoter and GFP reporter. Each of the five constructs was individually tested for ability to drive GFP expression in E5 chick neural tube with in ovo electroporation at E2 (**b**). CAG-DsRed construct was used as the transfection control. GFP expression was not observed in the sample electroporated with CR2.a^Δ95–96^ (**b**), which is the core region of the binding sites for Nkx6.1, Gsx1 and Phox2a/b (Table S2). Gene knockdown analysis was performed using shRNA constructs coupled with RFP reporter targeting Nkx6.1 or Phox2b ((**c**) and Fig. S2c). Each individual construct was electroporated individually into E2 chick neural tube together with CR2.a-GFP. A scramble shRNA sequence (scr-shRNA) was used as negative control (**c**). Chick embryos were harvested and examined for GFP and RFP expression at E5. The percentage of GFP+/RFP+ cells was determined by cell counting. A dramatic decrease of GFP+ cells was observed in samples with shNkx6.1 comparing to scr-shRNA (**c**). ChIP was performed to confirm the binding between transcription factors and CR2.a with spinal cord tissue harvested from E14.5 wild-type mouse embryos. Final DNA product was quantified by qRT-PCR with CR2.a primers. Significant precipitation was found with Nkx6.1 (C-terminus), H3K9Ac and Input Control compare to the negative control (ms IgG) using both percentage-input (**d**) and Fold-enrichment analysis (**e**). V, ventral; D, dorsal. Scale bars = 50 μm. T-test: **p-value < 0.01, ***p-value < 0.005; n = 3.

To identify the specific protein factors that interact with CR2.a, we performed site-directed mutagenesis on CR2.a sequence, followed by shRNA-mediated gene knockdown and chromatin immunoprecipitation (ChIP) analysis. Based on the core matrix of predicted TFBSs, 2–8 bps in the CR2.a sequence were deleted to eliminate the TFBSs of each TF clusters (Fig. 2a, Fig. S1 and Tables S2 and S6). The resulting five mutated sequences were examined with TFANSFAC^20^ to avoid the generation of new TFBSs. Then, the sequences were tested with the reporter assays in the chick neural tube at E2 together with a control CAG-DsRed construct, respectively (Fig. 2b). GFP expression was not observed in the samples electroporated with CR2.a^Δ95–96^ construct (Fig. 2b) compared to samples electroporated with CR2.a-GFP (Fig. 1d) or other mutated sequences (Fig. 2b). Three replicates of transfection with CR2.a^Δ95–96^ construct were performed and the results were consistent. This indicates that the 2 bp at 95^th^–96^th^ position from 5′ end of CR2 is essential for its enhancer activity. The deletion of these 2 bps in CR2 disrupts the core binding sites for Gsx1, Nkx6.1, Phox2a and Phox2b (Fig. 2a). Analysis of a published ChIP-seq result^21^ revealed that Phox2a does not bind to CR2 region. Since chick Gsx1 mRNA sequence were not available at the NCBI Nucleotide database, we thus further analyzed the remaining two factors Nkx6.1 and Phox2b for their ability to activate CR2.a using shRNA-mediated gene knockdown assay. The shRNA constructs for these two factors (shNkx6.1 and shPhox2b, with RFP as reporter, Table S4) were electroporated individually into the chick neural tube together with CR2.a-GFP at E2. The expression of GFP in the RFP+ cells was examined at E5 to determine the effect of knocking-down the factor on CR2.a activation. Results show that knockdown of Nkx6.1 dramatically decreased the number of GFP+ cells in RFP+ cell population, while knockdown of Phox2b has no effect on GFP expression (Fig. 2c).

To determine whether Nkx6.1 binds to CR2.a *in vivo* in mouse spinal cord, ChIP was performed with spinal cord tissue from E14.5 wild-type mouse embryos. qRT-PCR analysis of DNA products from ChIP showed that the antibody specific for C-terminus of Nkx6.1 can strongly precipitate CR2.a with 35-fold-larger enrichment compare to the background ms IgG (Fig. 2d,e). The antibody specific for N-terminus of Nkx6.1, however, does not precipitate with CR2.a (Fig. 2d,e). This distinct result from these two Nkx6.1 antibodies suggest that Nkx6.1 interact with CR2.a on its N-terminus. Furthermore, a high copy number of CR2.a was precipitated by an open chromatin marker H3K9Ac, confirming the regulatory activity of CR2.a (Fig. 2d,e). Together these data indicate that Nkx6.1 binds to CR2.a during the spinal cord development, and is essential to the regulatory function of CR2.a.

### Nkx6.1 regulates expression of Notch1 and neurogenesis-related genes

**Figure 3:**
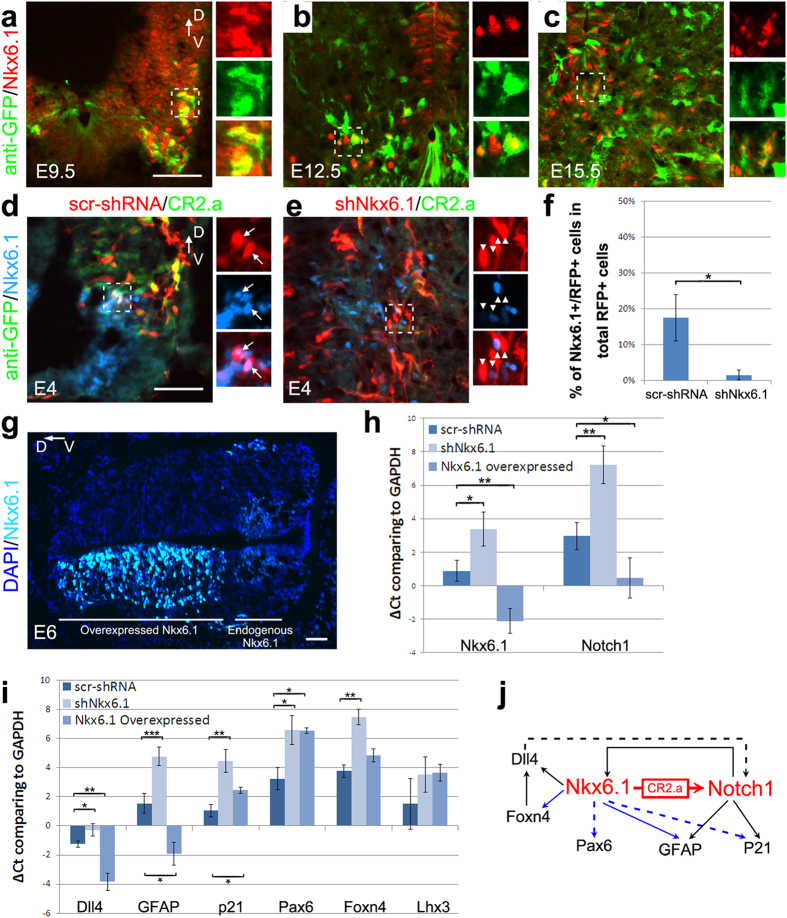
Nkx6.1 regulates Notch1 expression during early spinal cord development. The expression of Nkx6.1 and CR2-GFP in transgenic spinal cord was examined by immunofluorescence staining (**a–c**). GFP+ cells in the ventral spinal cord express Nkx6.1 at various stages from E9.5 to E15.5 (**a**–**c**). Loss- and gain-of-function assays were performed to determine the role of Nkx6.1 function on Notch1 expression using developing chick spinal cord model (**d–j**). Gene silencing (shNkx6.1-RFP) and overexpression constructs of Nkx6.1 was injected and electroporated into the neural tube at E2 and E5, respectively, to transfect the spinal cord tissues. As compared with scrambled-RNA control (**d**), number of CR2.a-GFP+ cells significantly decreased after Nkx6.1 knockdown (**e,f**), while overexpression of Nkx6.1 caused an ectopic expression of Nkx6.1 in the dorsal spinal cord (**g**). The level of gene expression was determined by qRT-PCR analysis of the transfected tissue. A larger ∆Ct (threshold cycle number) or a reduced Notch1 transcription was observed in samples with Nkx6.1 knockdown; while a smaller ∆Ct or an increased Notch1 transcription in samples with Nkx6.1 overexpression (**h**). The changes of Notch1 related genes Dll4, GFAP, p21, and interneuron related genes Pax6, Fxon4, and Lhx3 are shown in (**i**). A simplified gene regulatory network of Nkx6.1 and Notch1 is depicted in (**j**) for V2 interneuron development. Red and blue arrows represent data presented in this study. Black arrows represent data from literature review. Dotted lines indicate potential regulatory connections, while solid lines indicate connections with experimental verification. D, dorsal; V, ventral. Scale bars = 50 μm. T-test: *p-value < 0.05, **p-value < 0.01, ***p-value < 0.005; n = 3.

To determine the role of Nkx6.1 in regulating CR2.a activity, the expression of Notch1 and other neurogenesis-related genes, we first examined Nkx6.1 expression in CR2-GFP transgenic mouse by immunostaining on the sections from E9.5~E15.5 spinal cord. We found that GFP+ cells were co-labeled with Nkx6.1 (Fig. 3a–c) in the ventral spinal cord, supporting its role in regulating ventral domain specific Notch1 expression. Next, we examined whether Nkx6.1 knockdown affects the expression of Notch1 and neurogenesis-related genes. In the chick spinal cord, Nkx6.1 knockdown significantly reduced the number of both CR2.a-GFP+ cells (Figs 2g and 3d–e) and Nkx6.1+/RFP+ cells (Fig. 3d–f). Quantitative RT-PCR analysis revealed a significant reduction in the expression of Nkx6.1 and Notch1 (Fig. 3h, showing relative threshold cycle number (∆Ct) comparing to GAPDH). Transcription level of Notch1 was less than 1/16 (∆∆Ct > 4) of the control samples, while the level of Nkx6.1 is about 1/4 (∆∆Ct > 2). Such exponential decrease of Notch1 transcription level indicates that Nkx6.1 is required for proper enhancer activity during Notch1 expression. In addition, the transcription level of Notch1 downstream genes (e.g., GFAP and p21) and Notch1 associated gene (e.g., Dll4) was also found decreased in transfected cells (RFP+) after Nkx6.1 knockdown (Fig. 3i). We also found a decrease in the expression of some of the interneuron-related genes (e.g., Pax6 and Foxn4), while other genes (e.g., Lhx3) were not affected (Fig. 3i). Conversely, we overexpressed Nkx6.1 using plasmid Tet-O-FUW-Nkx6.1^19^ and analyzed its effect on the expression of Notch1 neurogenesis-related genes by qRT-PCR (Fig. 3g). Transfected cells showed an 8-fold increase in Nkx6.1 expression (∆∆Ct < −3) and a 4-fold increase in Notch1expression (∆∆Ct < −2) (Fig. 3h), suggesting that Nkx6.1 overexpression enhances Notch1 expression during spinal cord development. In addition, we observed increased transcripts for GFAP and Dll4, decreased transcripts for p21 and Pax6, and no significant changes in transcripts for Foxn4 or Lhx3 genes (Fig. 3i).

### CR2 activity is in neural stem/progenitor cells

**Figure 4:**
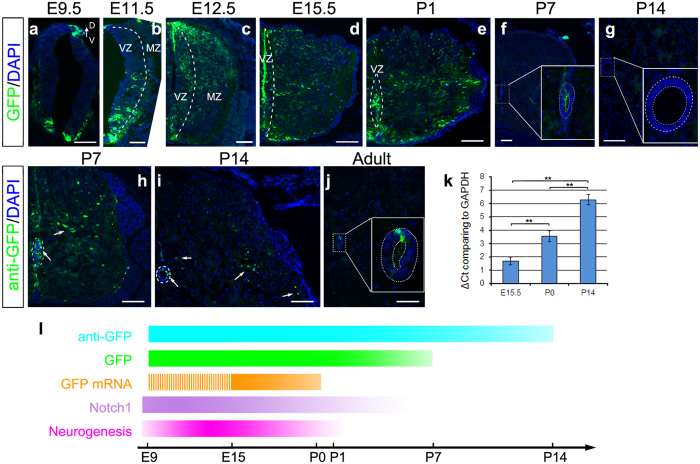
CR2 activity is parallel to neurogenesis in the developing spinal cord. GFP expression was examined in cross sections of CR2-GFP transgenic mouse spinal cord at various developmental stages, e.g., E9.5 (**a**), E11.5 (**b**), E12.5 (**c**), E15.5 (**d**), P1 (**e**), P7 (**f,h**), P14 (**g,i**), and adult (**j**). At E9.5, the majority of GFP+ cells were located in dorsal- and ventral-most regions of the developing spinal cord (**a**). At E11.5, GFP+ cells in the ventral spinal cord expanded to occupy the majority of ventral VZ and MZ (**b**). From E12.5 to P1, GFP+ cells in VZ remains, while other cells outside VZ were also found to be GFP+ (**c–e**). At P7 and P14, GFP+ cells can only be observed in the ependymal cells (enlarged in **f** and **g**). After P7, a low level of GFP can be detected by immunofluorescence staining with anti-GFP antibody (**h,i**). In adult, GFP+ cells can only be detected in ependymal cells surrounding the central canal (dotted circles in (**j**), also see Fig. 6). Arrows indicate regions where GFP+ cells are located. Dotted lines demonstrate the margin between VZ and MZ (**b–e**) and outlines the central canal (**f–j**). The level of GFP mRNA was determined by qRT-PCR and is shown in ∆Ct (threshold cycle number) (**k**), which is inversely correlated with the concentration of mRNA template. Timeline of GFP mRNA and protein expression is depicted along with Notch1 expression pattern and neurogenesis in the mouse spinal cord^26^ (**l**). VZ, ventricular zone; MZ, marginal zone; D, dorsal; V, ventral. Scale bars = 100 μm. **p-value < 0.01; n ≥ 3.

Next, we examined the spatiotemporal activity of CR2 in the CR2-GFP transgenic mouse spinal cord at various stages during embryonic and postnatal development. GFP reporter expression was first observed in the NSPCs at embryonic day (E) 9.5 near the roof plate (RP) and the floor plate (FP) (Fig. 4a). At E11.5, the GFP+ cells were mainly found in the ventral region of the ventricular zone (VZ) and marginal zone (MZ) (Fig. 4b, Fig. S3). Then at E12.5, the GFP in ventral MZ diminished and was found more in the dorsal region (Fig. 4c). From E15.5 to postnatal day (P) 1, a sub-group of cells in the VZ maintains high GFP intensity, while the cells outside the VZ expanded throughout the entire spinal cord and carried lower level of GFP (Fig. 4d,e). By P7, the number of GFP+ cells dramatically decreased; only a few ependymal cells near the central canal maintained a low level of GFP (Fig. 4f). No GFP+ cells were directly observed by P14 and later stages (Fig. 4g).

Since CR2 activity directly regulates GFP reporter expression, we thus analyzed the timing of the expression of GFP mRNA (Fig. 4k) and protein (with or without anti-GFP antibody staining) and compared with the known Notch1 expression (Fig. 4l). The timing of direct GFP detection and GFP mRNA expression are parallel to endogenous Notch1 expression, while the GFP signal retrieved by anti-GFP antibody staining can be detected by P14 (Fig. 4l). GFP protein has a relatively long half-life of ~26 hrs^22^. The GFP signal is visible for about seven days after its production^13^ and the remnant GFP protein in cells can be detected by immunohistochemistry for additional seven days (expression level reduce to <1%; calculation was based on half-life of 26 hrs). This allows us to trace the fate of GFP+ cells even when CR2 ceased its activity in the differentiated cells. With anti-GFP antibody staining, we were able to detect more GFP+ cells at P7 (Fig. 4h and Fig. S4g,h) and a few GFP+ cells outside ependymal cell layer at P14 (Fig. 4i). In the adult, GFP+ cells were only visible in the ependymal region with anti-GFP antibody staining (Fig. 4j). qRT-PCR analysis further showed that GFP mRNA level was decreased dramatically at P0 by comparison to the level at E15.5 and was even lower at P14 (Fig. 4k). Thus, CR2 activity was prominent in the NSPCs during embryonic stages and dramatically decreased by P0, which corresponds to the expression of Notch1 gene^23^,^24^,^25^ and the period of embryonic neurogenesis in mouse spinal cord^26^ (Fig. 4l).

### Cells once had CR2 activity preferentially became interneurons

**Figure 5:**
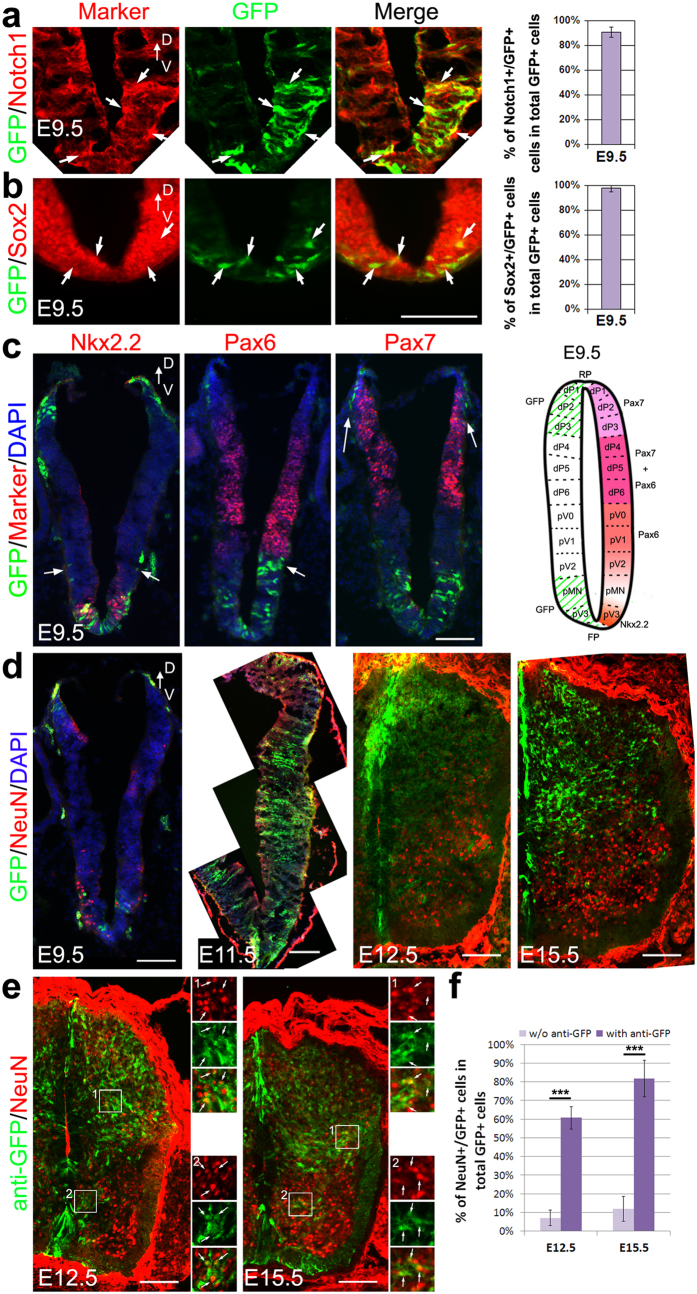
CR2 activity is prominent in neural progenitors during early embryonic spinal cord development. The identity of the CR2-GFP+ cells was examined in the transgenic spinal cord with immunofluorescence staining. GFP+ cells were stained with Notch1 (**a**) and neural stem cell marker Sox2 (**b**) at E9.5. Histograms show the quantification of the co-labeled GFP+ cells. Staining with the domain specific markers Nkx2.2, Pax6 and Pax7 (**c**) showed that at E9.5 GFP+ cells were localized to the dorsal progenitor layer 1–3 (dP1–3), motoneuron progenitor layer (pMN), and ventral progenitor layer 3 (pV3). Schematic drawing depicts the distribution of GFP+ cells in the E9.5 spinal cord. Staining of a post-mitotic neuronal marker NeuN showed little co-labeling with GFP from E9.5 to E15.5 (**d**), suggesting that CR2-GFP is not expressed in the differentiated neurons. However, the GFP+ progenitor cells differentiated into NeuN+ cells as suggested by cell-fate-tracing with anti-GFP antibody (**e**). Cell counting suggests a dramatic increase of GFP+/NeuN+ cells with anti-GFP tracing (**f**). Arrows indicate the co-labeled cells (**a,b,d,e**) or GFP+ cells (**c**). D, dorsal; V, ventral. Scale bars = 100 μm. T-test, ***p-value < 0.005; n ≥ 3.

The identities of GFP+ cells in the transgenic mice were determined by immunostaining with cell specific markers and anti-GFP. At E9.5, the vast majority of GFP+ cells were co-labeled with Notch1 (90.1 ± 4.3%; n = 3) and neural stem cell markers Sox2 (97.8 ± 3.2%; n = 3) (Fig. 5a,b). This confirms that GFP expression is in NSPCs. Using the progenitor domain specific markers Pax7 (dorsal progenitor layer 1–6 (dP1–6)), Pax6 (dP4–6 and the ventral progenitor layer 0–2 (pV0–2))^27^ and Nkx2.2 (pV3)^28^, we observed GFP expression in the dP1–3, pV3, and both dorsal and ventral region of the spinal cord (Fig. 5c). At later developmental stages, GFP started to diminish and only a few GFP+ cells were post-mitotic neurons as show by NeuN staining (Fig. 5d). However, with anti-GFP we were able to co-stain the GFP+ cells with NeuN (Fig. 5e), Brn3a and Pax2 (domain specific interneuron markers), Isl1 (a marker for dorsal layer 3 interneurons (dI3) and motoneurons) (Fig. S5), Evx1, En1, Chx10, Gata2 (ventral interneuron layer 0~2 (V0~2) markers) (Fig. S6), S100b, GFAP (glial/astrocyte markers) and Olig2 (oligodendrocyte marker) (Fig. S7). Compare with native GFP (without anti-GFP staining), we observed an increased percentage of NeuN labeled GFP+ cells from 81.3 ± 11.7% at E15.5 to 73.7 ± 8.1% at P1 (Fig. 5f), suggesting that the CR2-GFP+ cells differentiated into neurons. With Brn3a, 18.4 ± 7.8%, 23.6 ± 7.7% and 15.2 ± 1.7% of GFP+ cells were labeled in the dI1–3 and dI5 domain at E12.5, E15.5 and P1 (Fig. S5a–e). With Pax2, 33.8 ± 9.2%, 22.13 ± 4.7% and 26.1 ± 10.2% of GFP+ cells were labeled in the dI4 domain (Fig. S5d–f) while 7.8 ± 1.8%, 6.1 ± 3.4% and 4.7 ± 1.3% of GFP+ cells were labeled in the dI6 and V0–1 domains (Fig. S5g–i), respectively. Since the Pax2 staining of dI6 and V0–1 interneurons are clustered and cannot be distinguished, additional staining with V0 cell marker Evx1, V1 cell maker En1, and V2a cell marker Chx10, V2b cell marker Gata2 were performed (Fig. S6). The results showed that a small percentage of GFP+ cells reside in these ventral domains (Fig. S6). Meanwhile, immunostaining with Isl1 showed co-localization with GFP+ cells in dI3 but not in the differentiated motoneurons at E15.5 and later stages (Fig. S5). In addition, a few of GFP+ cells were co-stained with S100b, GFAP or Olig2 (Fig. S7), indicating that CR2 may not be active in developing astrocytes or oligodendrocytes. Our results show that the majority of the GFP+ cells reside in the Brn3 + and Pax2 + interneuron domains and not in mature motoneurons of the developing spinal cord (see summary distribution diagram in Fig. S8).

### CR2 activity persists in the adult NSPCs

**Figure 6:**
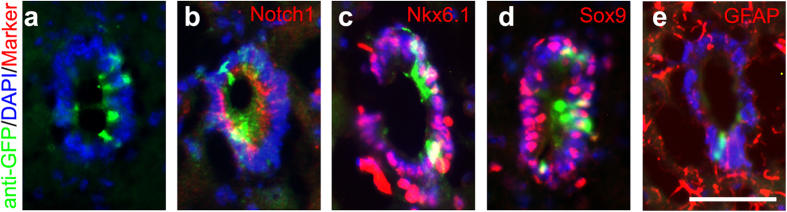
CR2 activity persists in the adult neural stem cells. Cross sections of adult transgenic mouse spinal cord (two to five-month old) were stained with anti-GFP antibody, Notch1, Nkx6.1, and adult stem cell marker Sox9 and GFAP. CR2-GFP+ cells were detected in a subset of the ependymal cells lining the central canal (**a**). GFP+ cells were co-labeled with Notch1 (**b**), Nkx6.1 (**c**), Sox9 (**d**) and GFAP (**e**). Scale bar = 50 μm.

Ependymal cells are known to be adult NSPCs, which remain quiescent in normal conditions and proliferate rapidly after injury^29^,^30^,^31^. A few GFP+ cells were detected in the ependymal cells lining the central canal (Figs 4j and 6a), suggesting CR2 activity is in the adult NSPCs. Double immunofluorescence staining shows that these GFP+ cells are positive for Notch1 (Fig. 6b) and Nkx6.1 (Fig. 6c). In addition, CR2-GFP+ cells were also positive for NSPC markers in the adult spinal cord^32^,^33^, e.g., Sox9 (Fig. 6d) and GFAP (Fig. 6e). Thus, CR2 is also involved in regulating gene expression in adult NSPCs.

## Discussion

**Figure 7:**
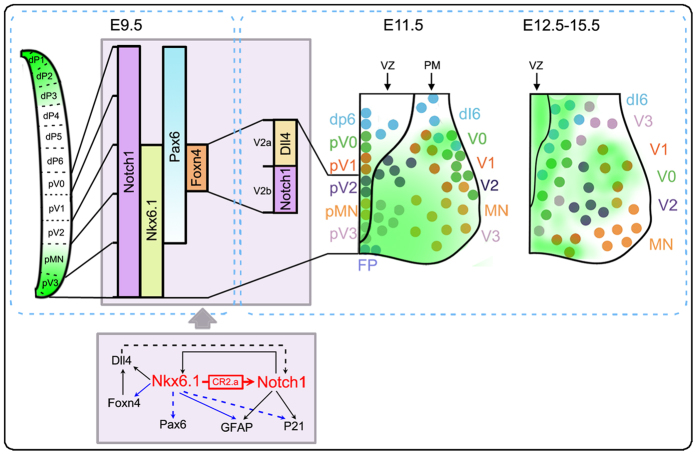
Model of gene regulation during ventral spinal cord development. Notch1 plays an important role during spinal cord development. Our data demonstrate that Nkx6.1 functions as a modulator for Notch1 expression in the ventral spinal cord. Nkx6.1 binds to CR2.a, a 139 bp enhancer in the 2^nd^ intron of Notch1, regulates the expression of Notch1, Notch1 downstream genes (e.g., GFAP and P21) and members of the regulatory network of the ventral spinal cord (e.g., Foxn4 and Dll4). The network centering Nkx6.1 and Notch1 is important for the differentiation of ventral spinal cord interneurons.

Our study in both chick and mouse models revealed a novel mechanism underlying Notch1 transcriptional regulation by the interaction of Nkx6.1 with Notch1 enhancer CR2 in NSPCs during ventral spinal cord development (Fig. 7).

### Nkx6.1 regulates the expression of Notch1 and affects genes involved in interneuron development in ventral spinal cord

Cell development in the spinal cord is controlled in a progenitor domain-specific manner. These domains are defined by the gradient expression of distinct factors, e.g., Nkx2.2/2.9/6.1/6.2, Pax3/6/7^27^,^34^. Notch1, a molecule critical for maintaining stem cell identity, is expressed throughout the ventricular zone of the developing spinal cord and is involved in fate determination of both dorsal and ventral interneurons^5^,^27^,^35^,^36^. The spatiotemporal activity of CR2 in transgenic mouse (Fig. 4 and 5) suggests that it is involved in the expression of Notch1 in NSPCs that are fated to become interneurons. Studies have shown that Nkx6.1 is expressed in pV2~V3 domains in the VZ during spinal cord development^15^ and both Notch1 and Nkx6.1 play a critical role in the V2 interneuron development^5^,^8^,^9^,^15^,^28^. Common effects were reported in the Notch1 and Nkx6.1 knockout animals, e.g., both animals showed an increase of V1 and V0 interneurons and a decrease of V2 interneurons and motoneurons^5^,^15^. Studies have also shown that there is a down-regulation of Nkx6.1 in Notch1 conditional knockout animals^5^. Our finding that Nkx6.1 directly regulates Notch1 expression in the ventral domains of developing spinal cord uncovers a feedback control mechanism between Notch1 and Nkx6.1 in NSPCs that fated to become ventral interneurons (Fig. 7). In addition, manipulation of Nkx6.1 expression affects several Notch1 down-stream genes and other critical genes for spinal cord development (Fig. 3). Previous studies have established that Notch1, Nkx6.1, Nkx2.2 and Olig2 constitute a gene regulatory network for the development of the ventral spinal cord^15^,^28^,^37^; Foxn4 and Pax6 are transcription factors both involved in the determination of V2 interneurons^14^,^34^; Dll4, a Notch ligand, is a marker for V2a interneuron^8^,^14^ and is regulated by Foxn4^38^. Our observation that the transcription level of Dll4, GFAP, p21, Foxn4 and Pax6 were all affected in the cells with Nkx6.1 knockdown (Fig. 3i) confirms the essential role of Nkx6.1 in regulating Notch1 signaling in ventral interneuron progenitor domains. The fact that the transcription levels of p21, Foxn4 and Pax6 were not changed significantly with Nkx6.1 overexpression implies a mechanism that compensates the effects of higher Nkx6.1 level in this network. The finding that Lhx3, a gene plays a role in V2a interneurons^14^, did not change its transcription level with Nkx6.1 knockdown may indicate that it is not a part of this Nkx6.1-Notch1 regulatory network (Fig. 3j). Thus, Nkx6.1 regulates Notch1 expression as part of the transcriptional regulatory network for the development of ventral interneurons originated from pV2~pV3. Furthermore, the lack of CR2-GFP expression in the middle VZ (e.g., pV0 and pV1 domains) (Fig. 4a) suggests Notch1 expression in this region is probably controlled by other regulatory elements and by other factors.

### Potential candidate regulator of Notch1 in dorsal spinal cord

In this study, we have shown Nkx6.1 binds to CR2 and regulates Notch1 expression during ventral spinal cord development. However, the regulation of Notch1 expression in the dorsal spinal cord remains unclear. CR2 is active in NSPCs of the dorsal spinal cord which develop into dorsal interneurons (Fig. 4). It is likely that the expression of Notch1 in dorsal spinal cord may also be regulated by CR2 via its interaction with a different transcription factor. Published studies have shown that Gsx1 and Nkx6.1 function in the spinal cord during similar period but in distinct domains, i.e., Gsx1 in the dorsal domain and Nkx6.1 in the ventral domain^15^,^35^,^39^. Our data also showed that Gsx1 is one of the candidate binding factor for CR2 (Fig. 2a,b). It is thus possible that the binding of Gsx1 with CR2 is a critical step in activating CR2 activity and Notch1 expression during dorsal spinal cord neurogenesis. The possibility that Gsx1 regulates CR2 dependent expression of Notch1 and subsequent Notch1 signaling in the developing dorsal spinal cord remains to be determined.

### The interaction of Nkx6.1with CR2 may also regulate Notch1 expression in the ependymal cells of adult spinal cord

Adult ependymal cells are originated from the Nkx6.1+/Gata2+/Gata3+ ventral spinal progenitors and maintain Nkx6.1 expression^40^,^41^. In consistent with these studies, we observed that CR2-GFP+ ependymal cells of adult spinal cord were positive for Nkx6.1 and Notch1 (Fig. 6b,c). It suggests a possibility that the interaction between CR2 and Nkx6.1 may also exist for Notch1 expression in the adult spinal cord. In addition, since CR2-GFP+ cells were only a subset of the adult ependymal cells (Fig. 6), it is likely that other enhancers may be involved in the regulation of adult NSPCs.

### Different functional minimum sequences of CR2 in the spinal cord and brain

We identified a 139 bp region (CR2.a) as a minimum sequence required for directing reporter gene expression in developing chick spinal cord (Fig. 1). Although a 18 bp piece of CR2 (86–104 bp in CR2) functions as an enhancer in the developing brain^13^, it lacks the ability to drive GFP expression in the spinal cord (Fig. 1b,d, CR2.d). This indicates that different mechanisms may be involved in the two different CNS tissues. CR2 may interact with a different group of nuclear factors in different parts of the CNS for its enhancer activity.

## Additional Information

**How to cite this article**: Li, Y. *et al*. Transcriptional Regulation of Notch1 Expression by Nkx6.1 in Neural Stem/Progenitor Cells during Ventral Spinal Cord Development. *Sci. Rep.* **6**, 38665; doi: 10.1038/srep38665 (2016).

**Publisher's note:** Springer Nature remains neutral with regard to jurisdictional claims in published maps and institutional affiliations.

## Supplementary Material

Supplementary Figures and Tables
