# Supplementary material for: Transcriptional Regulation of Notch1 Expression by Nkx6.1 in Neural Stem/Progenitor Cells during Ventral Spinal Cord Development
Source: Sci Rep. 2016 Dec 7;6:38665. doi: 10.1038/srep38665 (PMC5141430; doi:10.1038/srep38665)
Supplement: Supplementary Figures and Tables [file srep38665-s1.pdf]

SUPPLEMENTARY DATA

Transcriptional Regulation of Notch1 Expression by Nkx6.1 in Neural Stem/Progenitor Cells during Ventral Spinal Cord Development

Ying Li, Evangeline Tzatzalos, Kelvin Y. Kwan, Martin Grumet, and Li Cai *

*Corresponding author:

Li Cai, Ph.D.

Department of Biomedical Engineering

Rutgers University

599 Taylor Road

Piscataway, NJ 08854, USA

lcai@rutgers.edu

Tel: 848-445-6559

Fig. S1

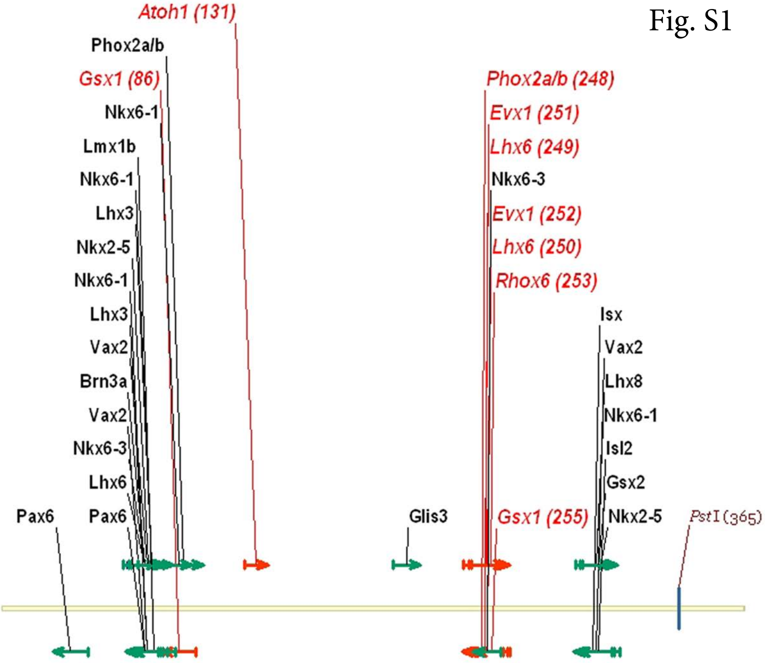
Atoh1 (131)
Phox2a/b
Gsx1 (86)
Nkx6-1
Lmx1b
Nkx6-1
Lhx3
Nkx2-5
Nkx6-1
Lhx3
Vax2
Brn3a
Vax2
Nkx6-3
Lhx6
Pax6
Pax6
Glis3
Phox2a/b (248)
Evx1 (251)
Lhx6 (249)
Nkx6-3
Evx1 (252)
Lhx6 (250)
Rhox6 (253)
Gsx1 (255)
Isx
Vax2
Lhx8
Nkx6-1
Isl2
Gsx2
Nkx2-5
PstI (365)

Fig. S2

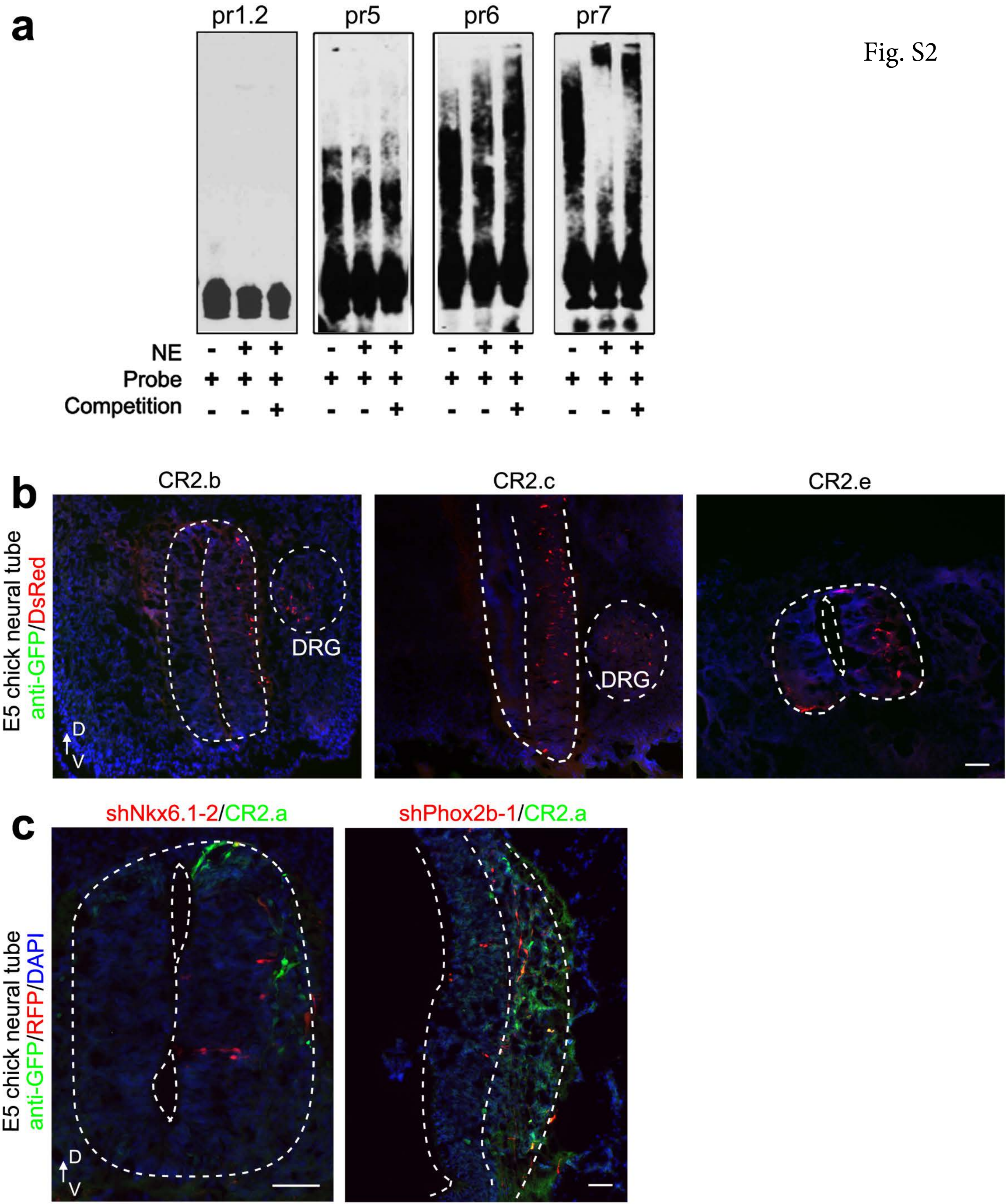
a
pr1.2
pr5
pr6
pr7
NE - + + - + + - + + - + +
Probe + + + + + + + + + + + +
Competition - - + - - + - - + - - +
b
CR2.b
CR2.c
CR2.e
E5 chick neural tube anti-GFP/DsRed
DRG
DRG
D
V
c
shNkx6.1-2/CR2.a
shPhox2b-1/CR2.a
E5 chick neural tube anti-GFP/RFP/DAPI
D
V

Fig. S3

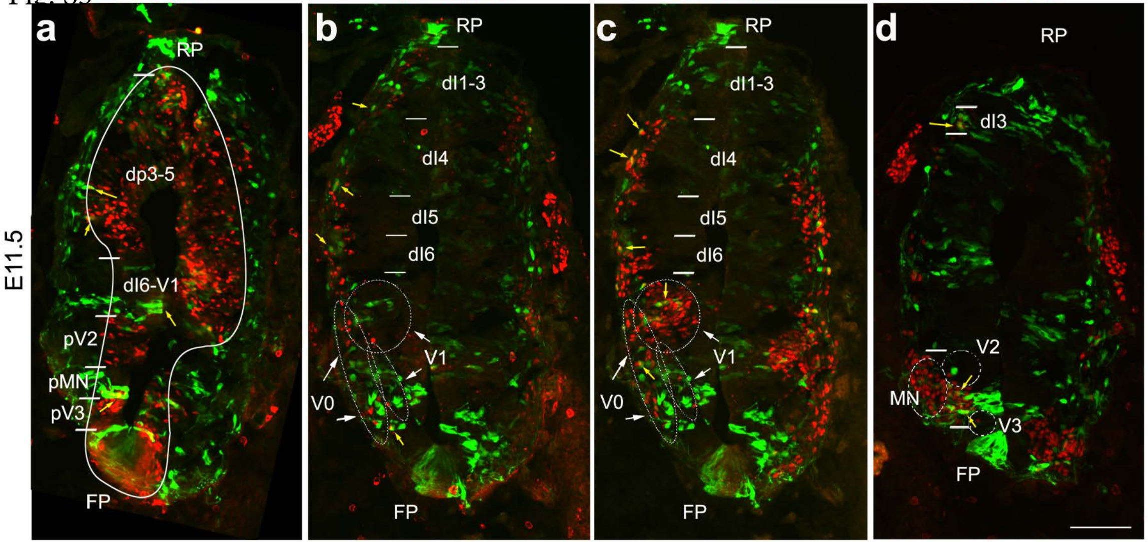

GFP/Ascl1
GFP/Brn3a
GFP/Pax2
GFP/Isl1
E11.5
a
RP
dp3-5
dI6-V1
pV2
pMN
pV3
FP
b
RP
dI1-3
dI4
dI5
dI6
V1
V0
FP
c
RP
dI1-3
dI4
dI5
dI6
V1
V0
FP
d
RP
dI3
V2
MN
V3
FP

Fig. S4

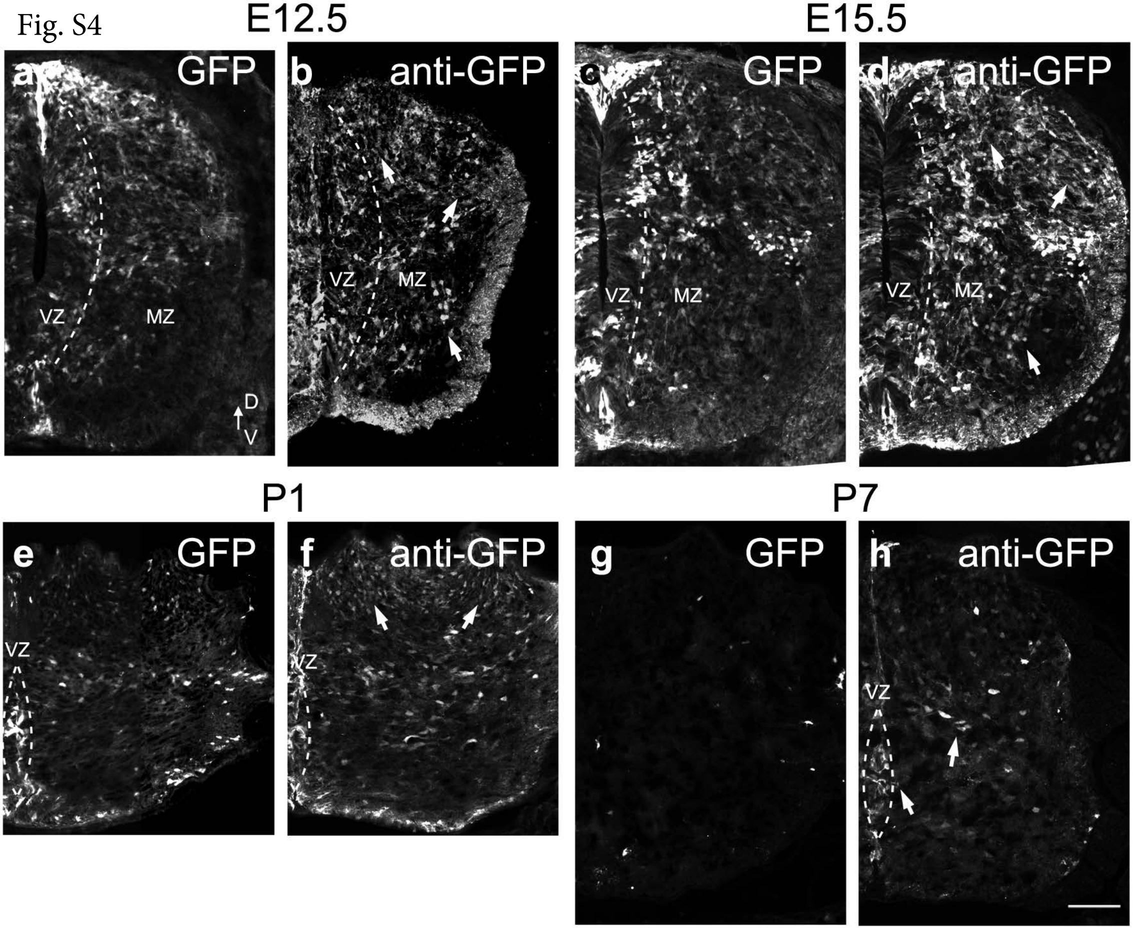

E12.5
E15.5
a
GFP
b
anti-GFP
c
GFP
d
anti-GFP
VZ
MZ
D
V
P1
P7
e
GFP
f
anti-GFP
g
GFP
h
anti-GFP

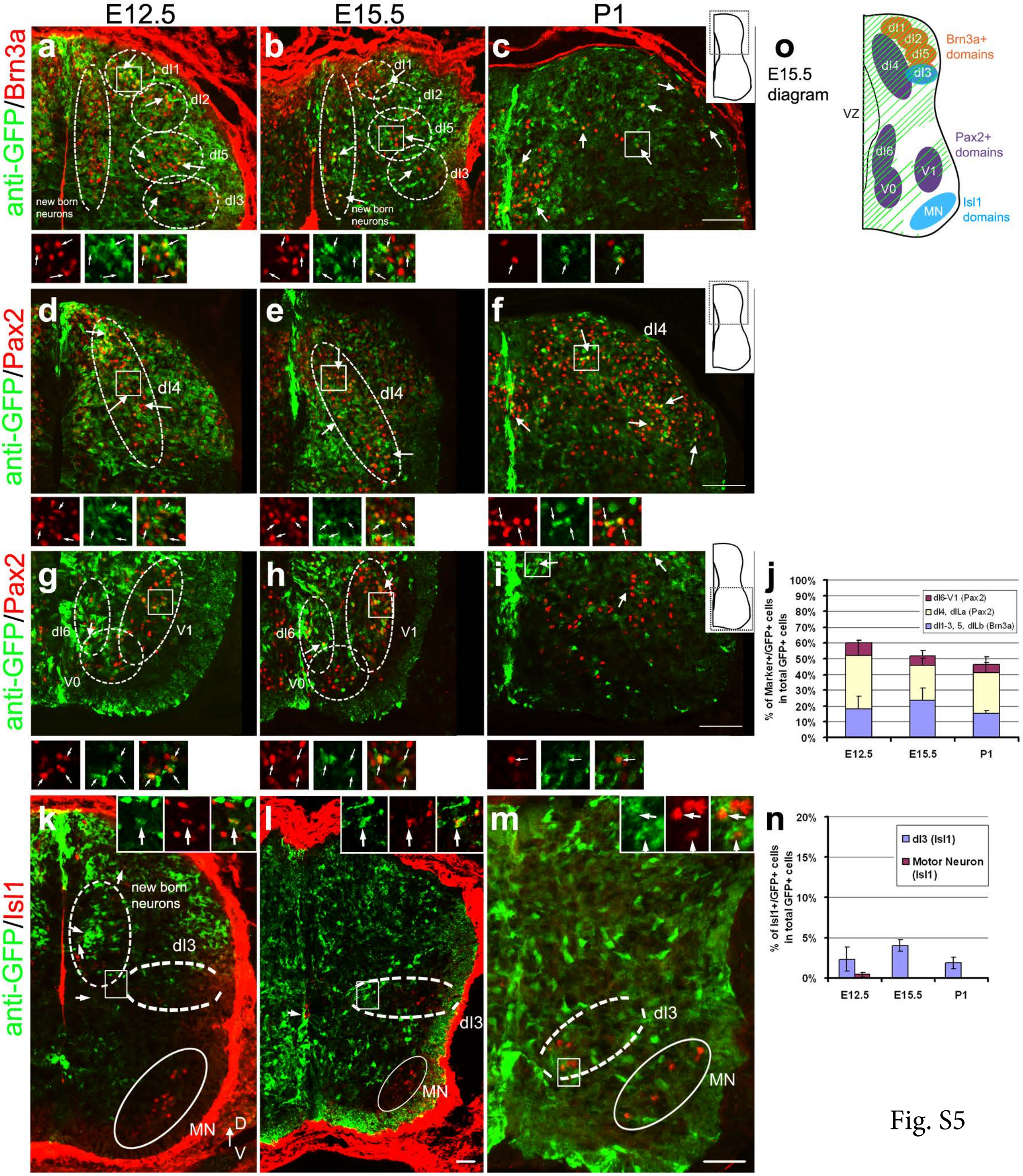
E12.5
E15.5
P1
anti-GFP/Brn3a
anti-GFP/Pax2
anti-GFP/Pax2
anti-GFP/Isl1
a
b
c
d
e
f
g
h
i
j
k
l
m
n
o
dI1
dI2
dI5
dI3
dI4
dI6
V0
V1
MN
new born neurons
D
V
E15.5 diagram
VZ
Brn3a+ domains
Pax2+ domains
Isl1 domains
% of Marker+/GFP+ cells in total GFP+ cells
dI6-V1 (Pax2)
dI4, dILa (Pax2)
dI1-3, 5, dILb (Brn3a)
100%
90%
80%
70%
60%
50%
40%
30%
20%
10%
0%
% of Isl1+/GFP+ cells in total GFP+ cells
dI3 (Isl1)
Motor Neuron (Isl1)
20%
15%
10%
5%
0%
E12.5
E15.5
P1

Fig. S5

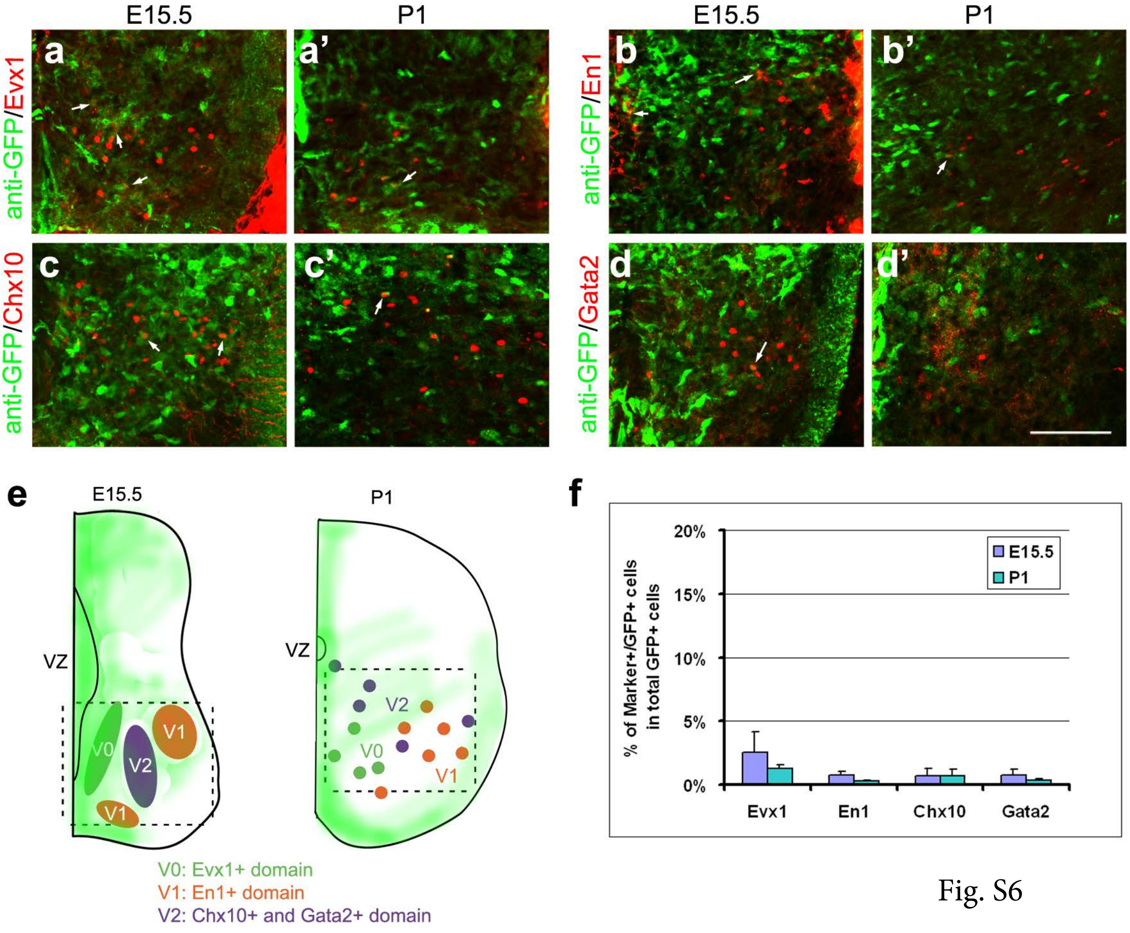

E15.5
P1
E15.5
P1
anti-GFP/Evx1
anti-GFP/En1
anti-GFP/Chx10
anti-GFP/Gata2
a
a'
b
b'
c
c'
d
d'
e
E15.5
P1
VZ
V0
V1
V2
VZ
V2
V0
V1
V0: Evx1+ domain
V1: En1+ domain
V2: Chx10+ and Gata2+ domain
f
% of Marker+/GFP+ cells in total GFP+ cells
20%
15%
10%
5%
0%
E15.5
P1
Evx1
En1
Chx10
Gata2

Fig. S6

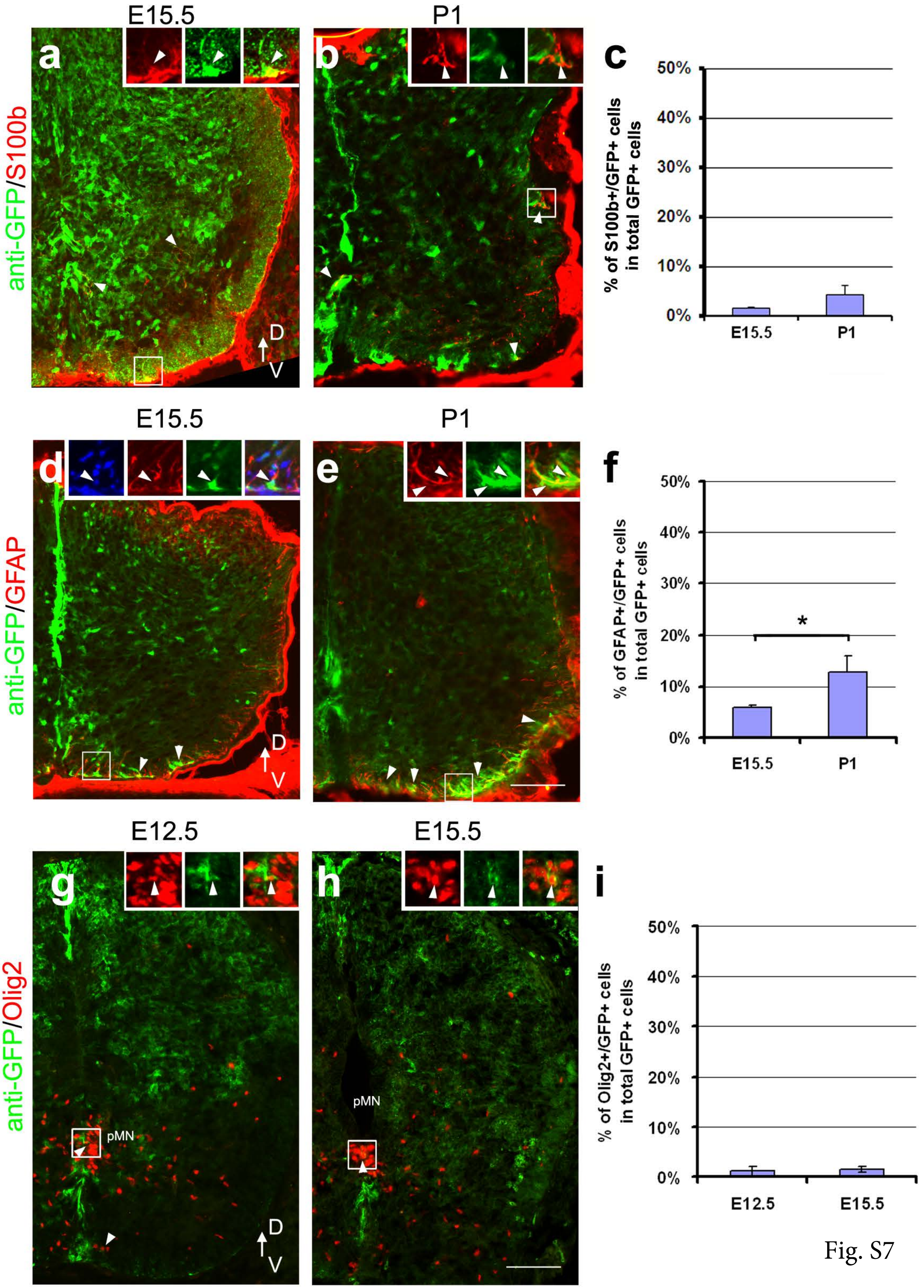
E15.5
P1
a
b
c
anti-GFP/S100b
D
V
50%
40%
30%
20%
10%
0%
% of S100b+/GFP+ cells in total GFP+ cells
E15.5
P1
E15.5
P1
d
e
f
anti-GFP/GFAP
D
V
% of GFAP+/GFP+ cells in total GFP+ cells
*
E15.5
P1
E12.5
E15.5
g
h
i
anti-GFP/Olig2
pMN
pMN
D
V
% of Olig2+/GFP+ cells in total GFP+ cells
E12.5
E15.5

Fig. S7

Fig. S8

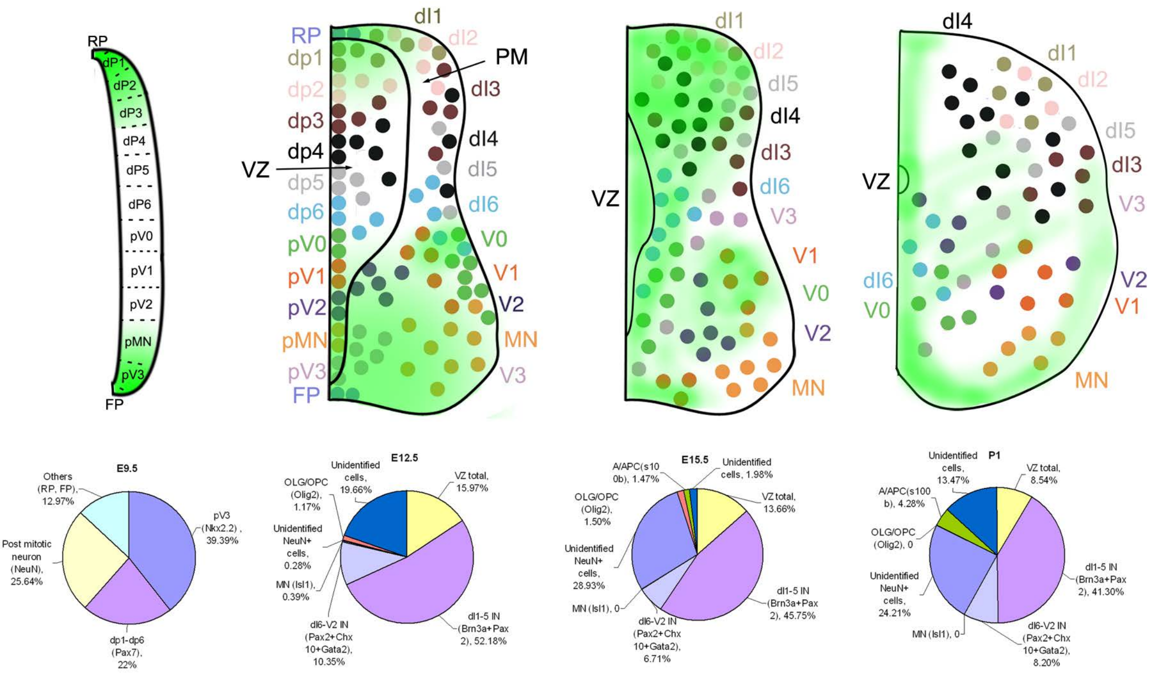
E9.5
E11.5
E12.5-15.5
P1-7
RP
dP1
dP2
dP3
dP4
dP5
dP6
pV0
pV1
pV2
pMN
pV3
FP
RP
dp1
dp2
dp3
dp4
VZ
dp5
dp6
pV0
pV1
pV2
pMN
pV3
FP
dI1
dI2
PM
dI3
dI4
dI5
dI6
V0
V1
V2
MN
V3
dI1
dI2
dI5
dI4
dI3
dI6
V3
V1
V0
V2
MN
VZ
dI4
dI1
dI2
dI5
dI3
V3
VZ
V2
V1
dI6
V0
MN
E9.5
Others (RP, FP), 12.97%
pV3 (Nkx2.2), 39.39%
Post mitotic neuron (NeuN), 25.64%
dp1-dp6 (Pax7), 22%
E12.5
Unidentified cells, 19.66%
OLG/OPC (Olig2), 1.17%
VZ total, 15.97%
Unidentified NeuN+ cells, 0.28%
MN (Isl1), 0.39%
dI6-V2 IN (Pax2+Chx 10+Gata2), 10.35%
dI1-5 IN (Brn3a+Pax 2), 52.18%
E15.5
A/APC(s10 0b), 1.47%
Unidentified cells, 1.98%
OLG/OPC (Olig2), 1.50%
VZ total, 13.66%
Unidentified NeuN+ cells, 28.93%
MN (Isl1), 0
dI6-V2 IN (Pax2+Chx 10+Gata2), 6.71%
dI1-5 IN (Brn3a+Pax 2), 45.75%
P1
Unidentified cells, 13.47%
VZ total, 8.54%
A/APC(s100 b), 4.28%
OLG/OPC (Olig2), 0
Unidentified NeuN+ cells, 24.21%
dI1-5 IN (Brn3a+Pax 2), 41.30%
MN (Isl1), 0
dI6-V2 IN (Pax2+Chx 10+Gata2), 8.20%

Supplemental Figure Legends

Figure S1. Potential transcription factor binding sites (TFBS) on CR2.
A schematic representation of the predicted TFBSs and their positions on the 399 bp CR2 sequence in the second intron of Notch1 (yellow bar). Arrows represent TFBSs and their relative positions on CR2. These 20 factors have expression in the spinal cord during embryonic and/or neonatal stages. Of the TFBSs of these factors, nine (TF names and arrows in red) are 100% conserved between mouse and chicken.

Figure S2. Nuclear protein binding and functional assays of CR2 subregions. A total of 10 probes and five sub-regions were designed based on the TFBS analysis (**Fig. 1a**). Addition results are shown here. In EMSA, pr1.2, pr5, pr6 and pr7 showed no bands (**a**). In reporter assay, subregions CR2.b, 2.c and 2.e did not allow GFP expression in developing chick neural tube (**b**). In gene knockdown assay, two sets of shRNA targeting each of Nkx6.1 and Phox2b were tested. Results of one set of constructs are shown in **Fig. 2**, and the 2nd set of constructs are shown here (**c**). NE, nuclear extract; DRG, dorsal root ganglion; D, dorsal; V, ventral. Scale bars = 50μm.

Figure S3. CR2 activity is found mainly in ventral spinal cord at E11.5. Staining with domain specific markers was performed to determine the identity of CR2-GFP+ cells at E11.5, including dp3~5 marker Ascl1 (**a**), dI1~3, 5 and V0 marker Brn3a (**b**), dI4 and dI6~V1 marker Pax2 (**c**), dI3 and MN marker Isl1 (**d**). GFP was found mainly in the ventral ventricular zone (pV0~3, pMN) and ventral marginal zone. Some GFP+ cells were also found in the dorsal marginal zone. VZ, ventricular zone; MZ, marginal zone; RP, roof plate; FP, floor plate; dp, dorsal interneuron progenitor layer; dI, dorsal interneuron layer; pV, ventral interneuron progenitor layer; V, ventral interneuron layer; pMN, motorneuron progenitor layer; MN motorneuron layer. Scale bars = 100μm.

Figure S4. Comparison of GFP expression in the transgenic mice with or without antibody retrieval. GFP expression in transgenic spinal cord at various developmental stages, e.g., E12.5 (**a-b**), E15.5 (**c-d**), P1 (**e-f**), and P7 (**g-h**) were examined by direct observation of GFP fluorescence (**a**, **c**, **e**, **g**) and with anti-GFP antibody staining (**b**, **d**, **f**, **h**). In all four stages, antibody staining either enhanced the GFP signal or revealed more cells have or had GFP expression. Arrows indicate cells only visualized in samples with anti-GFP staining. VZ, ventricular zone; MZ, margin zone. Scale bars = 100 μm.

Figure S5. CR2 activity is preferentially in interneuron progenitor domains but not motoneuron domain. Cross sections of transgenic spinal cord at E12.5, E15.5 and P1 were stained with domain-specific markers Brn3a (**a-c**), Pax2 (**d-i**) (interneuron) and Isl1 (**k-m**) (interneuron and motoneuron). Co-labeled GFP+ cells (arrows) were found in all Brn3a+ domains, e.g., the region adjacent to the ventricular zone and the dorsal interneuron layers 1-3 and 5 (dI1-3, dI5) (dashed oval circles in **a-c**). Co-labeled GFP+ cells were also found in all Pax2+ domains including the dorsal interneuron layer 4, 6 (dI4, dI6), the ventral interneuron layer 0-1 (V0-1) (dashed circles/ovals in **d-i**) and Isl1+ dI3 domain (dashed ovals in **k-m**). No Isl1+/GFP+ cells were found in the motoneuron domain (MN) (solid ovals in **k-m**). Boxed regions are shown in higher magnification with arrows indicating co-labeled cells. Histogram shows the quantification of Brn3a+/GFP+, Pax2+/GFP+ and Isl1+/GFP+ cells (**j, n**). Less than 0.5% co-labeled cells were found in MN layer at E12.5 and 0% at E15.5 and P1 (**n**). Schematic of the distribution of Brn3a+ (orange), Pax2+ (purple) and Isl1+ (blue) cells at E15.5 (**o**). Green shaded area indicates the expression pattern of GFP. D, dorsal; V, ventral. Scale bars = 100 μm; $n \geq 3$.

Figure S6. A small portion of CR2-GFP+ cells are located in the V0-2 domains of the developing spinal cord. Cross sections of transgenic spinal cord at E15.5 and P1 were stained with domain specific markers Evx1, En1, Chx10 and Gata2 (**a-d**). The expression pattern of the four markers is shown in schematic diagrams (**e**, with the dotted rectangle areas shown in (**a-d**)). A histogram of the marker+/GFP+ cells shows that about 3% of GFP+ cells locate in V0 at E15.5 and about 1% at P1, and less than 1% of GFP+ cells locate in V1 or V2 at E15.5 and P1 (**f**). Scale bar = 100 μm.

Figure S7. A small portion of CR2-GFP+ cells express S100b, GFAP and Olig2.

Cross sections of transgenic spinal cord at various developmental stages were stained with the astrocyte marker S100b (**a, b**), GFAP (**d, e**) and oligodendrocyte progenitor/oligodendrocyte marker Olig2 (**g-h**). S100b+/GFP+ (co-labeled) cells were found in both the grey matter and white matter at E15.5 (**a**, arrowheads). At P1, a few co-labeled cells can only be found in the white matter (**b,** arrowheads). Quantification shows the percentages of GFP+/S100b+ cells are small (**c**). GFAP+/GFP+ co-labeled cells were found in white matter at both E15.5 and P1 (**d**, **e**, arrowheads). Quantification shows the percentages of GFAP+/GFP+ cells increased from E15.5 to P1 (**f**). Olig2+/GFP+ cells were found at both E12.5 and E15.5, but only in the pMN domain where oligodendrocyte progenitor originates (**g**, **h**, arrowheads). No co-labeled cell can be found outside the ventricular zone. Quantification shows the percentage of co-labeled cells is about 1% (**i**). Boxed regions are shown in higher magnification with arrowheads indicating co-labeled cells. D, dorsal; V, ventral. Scale bar = 100 µm. T-test: * p-value < 0.05, ** p-value < 0.01; $n \geq 3$.

Figure S8. Composition and distribution of CR2-GFP+ cells at various stages during spinal cord development. Schematic diagrams of the developing spinal cord at various stages. Pie charts summarize the pattern of GFP expression in embryonic and neonatal transgenic mice, respectively. Colored coded circles represent cells from different progenitor domains of the spinal cord. The green shades indicate regions with GFP expression. RP, roof plate; FP, floor plate; dP1-6, dorsal progenitor layer 1-6; pV0-3, ventral progenitor layer 0-3; pMN, motor neuron progenitor layer; dI1-6, dorsal interneuron domain 1-6; V0-3, ventral interneuron 1-3; MN, motor neuron domain/motor neuron; VZ, ventricular zone; OLG, oligodendrocyte; OPC, oligodendrocyte progenitor cell; A, astrocyte; APC, astrocyte progenitor cell; IN, interneuron.

Supplemental Tables

Table S1. Construct design of CR2 subregions for *in ovo* electroporation and reporter assay.

Name	Region (bp)	Sequence/Primer sequence
CR2.a	52~190	Forward: TGTACATTCTGGGAAGCCACGCAT
		Reverse: TCACGGACGCTTGATGCCCTAAAT
CR2.b	162~341	Forward: ATAAGATTTAGGGCATCAAGCGTCCG
		Reverse: CCCGCCCTGTACTGAAGCCAATTA
CR2.c	52-91	TGTACATTCTGGGAAGCCACGCATAATTAATCACACAGCA
CR2.d	80-114	AATCACACAGCATTAATCGCCTCCCAACAATAGCT
CR2.e	115-164	GCTGCCCTTCTACTGAATCCCAGCTGTCGGCCTCTGAATGGA AGGA

Table S2. Primer design of CR2.a for mutagenesis assay and TFBS analysis.

Name	Primer sequence	Transcription factor binding sites (TFBS) involved
CR2.a$^{\Delta 76\text{-}79*}$	F:TGGGAAGCCACGCATAATCACACAGCATTA	Lhx6, Lhx3, Prrx2, Vax2, Nkx6.3, Nkx2.5, Nkx6.1, Pou4f1/2/3, Pax6
	R:TAATGCTGTGTGATTATGCGTGGCTTCCCA	
CR2.a$^{\Delta 75\text{-}82*}$	F:TGGGAAGCCACGCACACACAGCATTA	Lhx6, Lhx3, Prrx2, Vax2, Nkx6.3, Nkx2.5, Nkx6.1, Pou4f1/2/3, Pax6
	R:TAATGCTGTGTGTGCGTGGCTTCCCA	
CR2.a$^{\Delta 91\text{-}94}$	F:CGCATAATTAATCACACAGCATCGCCTCCCAACAATAGCTGCTG	Lhx3, Lmx1b, Nkx6.1, Gsx1, Phox2a/b
	R:CAGCAGCTATTGTTGGGAGGCGATGCTGTGTGATTAATTATGCG	
CR2.a$^{\Delta 95\text{-}96}$	F:CATAATTAATCACACAGCATTACGCCTCCCAACAATAGCTGCTG	Nkx6.1, Phox2a/b, Gsx1
	R:CAGCAGCTATTGTTGGGAGGCGTAATGCTGTGTGATTAATTATG	
CR2.a$^{\Delta 136\text{-}139}$	F:CTGCTGCCCTTCTACTGAATCCCGTCGGCCTCTGAATGGAAG	Atoh1
	R:CTTCCATTCAGAGGCCGACGGGATTCAGTAGAAGGGCAGCAG	

* these two deletion constructs were generated to eliminate TFBSs for Gsx1 and Nkx6.1 on CR2.a.

Table S3. Probe design for EMSA

Probes	Location (bp)	Sequence (forward strand)
pr1	61-114	TGGGAAGCCACGCATAATTAATCACACAGCATTAAT CGCCTCCCAACAATAGCT
pr2	115-170	GCTGCCCTTCTACTGAATCCCAGCTGTCGGCCTCTGA ATGGAAGGAAATAAGATTT
pr3	230-287	GCAAAAAAAAAAAAAAAGTAGTGTGCATTCATTAGT GTCTGACAGAGGCACAATCGGC
pr4	171-229	AGGGCATCAAGCGTCCGTGAGGCTTCTGCAAGGGGG AGAAAAGGCCCCCCCCAAAAAAA
pr5	288-340	TTTGTCCAATAAACTGCTCACAGACCTGCTTAATTGG CTTCAGTACAGGGCGG
pr6	341-399	GCCAGCCAGGGAGGTGGGGCTGCAGCCCACAGGCTG GGTACTGGAGGCAGCAGCACCCG
pr7	1-60	CCCTGCCAGTGAGGCAGGCAGACCAAGGAGCACAG AGGCGAGGAAGGGGGTTGTACATTC
pr1.1	60-92	CTGGGAAGCCACGCATAATTAATCACACAGCA
pr1.2	80-100	AATCACACAGCATTAATCGCC
pr1.3	80-114	AATCACACAGCATTAATCGCCTCCCAACAATAGCT
pr2.1	125-148	TACTGAATCCCAGCTGTCGGCCTC

Table S4. Sequence design of shRNA targeting Nkx6.1 and Phox2b factors in chick.

Name	Sequence
shNkx6.1-1F	GATCCGAGGACGACGACGACGACTACAACAATCAAGAGTTGTTGTAGTCGTCGTCGTCGTCCTCTTTTTGGAAA
shNkx6.1-1R	AGCTTTTCCAAAAAGAGGACGACGACGACTACAACAACTCTTGATTGTTGTAGTCGTCGTCGTCGTCCTCG
shNkx6.1-2F	GATCCGGAGAAGACTTTCGAGCAGACCATCAAGAGTGGTCTGCTCGAAAGTCTTCTCCTTTTTGGAAA
shNkx6.1-2R	AGCTTTTCCAAAAAGGAGAAGACTTTCGAGCAGACCACTCTTGATGGTCTGCTCGAAAGTCTTCTCCG
shPhox2b-1F	GATCCGCTTCCAGTATAACCCCATAAGGACCACTTCAAGAGAGTGGTCCTTATGGGGTTATACTGGAAGCTTTTTGGAAA
shPhox2b-1R	AGCTTTTCCAAAAAGCTTCCAGTATAACCCCATAAGGACCACTCTCTTGAAGTGGTCCTTATGGGGTTATACTGGAAGCG
shPhox2b-2F	GATCCGGAGACGCACTACCCCGACATTTACATCAAGAGTGTAAATGTCGGGGTAGTGCGTCTCCTTTTTGGAAA
shPhox2b-2R	AGCTTTTCCAAAAAGGAGACGCACTACCCCGACATTTACACTCTTGATGTAAATGTCGGGGTAGTGCGTCTCCG

Table S5. qRT-PCR primers

Name	Sequence
For mouse	
GAPDH-F	5’- ACTCTTCCACCTTCGATGCCG -3’
GAPDH-R	5’- CCGAGTTGGGATAGGGCCTC -3’
GFP-F	5’- CTCGTGACCACCCTGACCTA -3’
GFP-R	5’- CTTGTAGTTGCCGTCGTCCT -3’
Notch1-F	5’- ACAGTGCAACCCCCTGTATG -3’
Notch1-R	5’- AGTTGTTCCGTAGCTGGTCG -3’
For chick	
GAPDH-F	5’- TCAAATGGGCAGATGCAGGT -3’
GAPDH-R	5’- GATGGCATGGACAGTGGTCA -3’
Notch1-F	5’- CAACTGCCAGAACTTGGTGC -3’
Notch1-R	5’- AGAAAGGGCTGCAGTCATCC -3’
Nkx6.1-F	5’- GTCGCTCGTCTCACCTCAC -3’
Nkx6.1-R	5’- TGCCACGCTTTTTCAAGACG -3’
Pax6-F	5’- CCCACCATGCAGAACAGTCA -3’
Pax6-R	5’- CAGACCCCCTCCGAGAGTAA -3’
Lhx3-F	5’- CTGACTACGAGACGGCCAAG -3’
Lhx3-R	5’- GGGCTCGTCTGTGAAGGAGA -3’
GFAP-F	5’- TGGAGAGGTGATCAAGGAGTC -3’
GFAP-R	5’- ATCAACCTGCCTCCCCCTAT -3’
p21-F	5’- ACGAGCAGATCCAGAACGAC -3’
p21-R	5’- TTGGAGCCGTAGAAGTCTTTGA -3’
Foxn4-F	5’- CTGGATAGCTACTGCGTGCG -3’
Foxn4-R	5’- GGGCTGTCTTGAAGTAGGGAAA -3’
Dll4-F	5’- GGGCTGTGTTGTGTTCCTTTG -3’
Dll4-R	5’- GAACGACAGATGCACTCTCCA -3’

Table S6. Known function of the 13 transcription factors with binding sites on CR2.a.

Transcription factor	Binding site(s) (bp)	Expression and Functions	References
Pax6	70-84	Expresses during early neurogenesis and defines the dP4-pV2 progenitor domains in the ventricular zone of spinal cord.	(Ericson et al., 1997; Helms and Johnson, 2003; Liem et al., 2000)
Brn3a	68-86	Expresses both in the pre- and post-mitotic interneurons and defines the dI1-3, dI5 and V0 domains	(Caspary and Anderson, 2003; Helms and Johnson, 2003)
Lhx6	66-88	Expresses in dorsal spinal cord during early neurogenesis. Function in spinal cord is unknown but it mediates the interneuron fate determination and migration during brain development.	(Alifragis et al., 2004; Choi et al., 2005; Gong et al., 2003)
Nkx6.3	70-84	Expresses in ventral spinal cord at E13.5. Function in spinal cord is unknown but it can promote V2 IN production in hindbrain.	(Gray et al., 2004; Hafler et al., 2008)
Vax2	68-86, 69-87	mRNA was found expressed in spinal cord. Function in spinal cord is unknown but it is can define the ventral side of retina.	(Barbieri et al., 1999; LeDoux et al., 2006)
Lhx3	67-89, 70-92	Defines the MN, V2 progenitor domains.	(Sharma et al., 1998; Zhadanov et al., 1995)
Nkx6.1	71-85, 74-88, 88-102	Defines the MN, V2-3 progenitor domains.	(Liem et al., 2000; Sander et al., 2000)
Nkx2.5	70-88	Expresses in brain and cervical spinal cord. No known function in CNS.	Eurexpress (http://www.eurexpress.org/)
Lmx1b	71-93	Defines dI5/dILB progenitor domains. It is required for terminal differentiation of neurons locate in the uppermost layers of dorsal horn.	(Ding et al., 2004)
Gsx1	86-104	Expresses in dI3-5 and dIL progenitors at E10.5-E13.5.	(Mizuguchi et al., 2006; Valerius et al., 1995)
Phox2a/b	88-108	Phox2a co-stain with Lmx1b at E11.5. Forced expression of Phox2b will promote neuronal differentiation and projection.	(Ding et al., 2004; Dubreuil et al., 2000; Pattyn et al., 1997)
Atoh1	131-143	It is involved in dp1/dI1 domain specification in which cells will later develop into commissural interneurons.	(Bermingham et al., 2001; Helms and Johnson, 1998)

Table S7. Predicted transcription factor binding sites on CR2

Transcription factor <-> matrix family assignment				Position			Strand
Matrix Family	Detailed Family Information	Matrix	Detailed Matrix Information	from	to	anchor	
V$EREF	Estrogen response elements	V$ER.03	Estrogen response elements, IR3 sites	6	28	17	(+)
V$SMAD	Vertebrate SMAD family of transcription factors	V$SMAD.01	Sma- and Mad-related proteins	16	26	21	(-)
V$PAX5	PAX-2/5/8 binding sites	V$PAX5.01	B-cell-specific activator protein	23	51	37	(+)
V$PAX6	PAX-4/PAX-6 paired domain binding sites	V$PAX6.04	PAX6 paired domain binding site	28	46	37	(-)
V$PAX9	PAX-9 binding sites	V$PAX9.01	Zebrafish PAX9 binding sites	29	49	39	(+)
V$PLAG	Pleomorphic adenoma gene	V$PLAG1.01	Pleomorphic adenoma gene (PLAG) 1, a developmentally regulated C2H2 zinc finger protein	33	55	44	(+)
V$E2FF	E2F-myc activator/cell cycle regulator	V$E2F1_DP1.01	E2F-1/DP-1 heterodimeric complex	33	49	41	(+)
V$ETSF	Human and murine ETS1 factors	V$ETV1.02	Ets variant 1	34	54	44	(+)
V$KLFS	Krueppel like transcription factors	V$BTEB3.01	Basic transcription element (BTE) binding protein, BTEB3, FKLF-2	40	56	48	(+)
V$PLAG	Pleomorphic adenoma gene	V$PLAG1.02	Pleomorphic adenoma gene 1	44	66	55	(+)
V$STAF	Selenocysteine tRNA activating factor	V$STAF.01	Se-Cys tRNA gene transcription activating factor	46	68	57	(-)
V$FAST	FAST-1 SMAD interacting proteins	V$FAST1.01	FAST-1 SMAD interacting protein	47	63	55	(+)
V$HEAT	Heat shock factors	V$HSF1.01	Heat shock factor 1	49	73	61	(-)
V$STAT	Signal transducer and activator of transcription	V$STAT5.01	STAT5: signal transducer and activator of transcription 5	52	70	61	(-)
V$BCL6	POZ domain zinc finger expressed in B-Cells	V$BCL6.04	B-cell CLL/lymphoma 6, member B (BCL6B)	53	69	61	(-)
V$STAT	Signal transducer and activator of transcription	V$STAT.01	Signal transducers and activators of transcription	54	72	63	(+)
V$AHRR	AHR-arnt heterodimers and AHR-related factors	V$AHRARNT.02	Aryl hydrocarbon / Arnt heterodimers, fixed core	59	83	71	(-)
V$PAX5	PAX-2/5/8 binding sites	V$PAX5.02	B-cell-specific activator protein	60	88	74	(-)
V$CART	Cart-1 (cartilage homeoprotein 1)	V$S8.01	Binding site for S8 type homeodomains	65	85	75	(-)
V$SORY	SOX/SRY-sex/testis determinig and related HMG box factors	V$HMGA.01	HMGA family of architectural transcription factors (HMGA1, HMGA2)	66	90	78	(-)

V$DLXF	Distal-less homeodomain transcription factors	V$DLX1.01	DLX-1, -2, and -5 binding sites	66	84	75	(+)
V$LHXF	Lim homeodomain factors	V$LHX6.01	LIM homeobox 6	66	88	77	(+)
V$PDX1	Pancreatic and intestinal homeodomain transcription factor	V$IPF1.01	Insulin promoter factor 1, pancreatic and duodenal homeobox 1 (Pdx1)	66	84	75	(+)
V$OCT1	Octamer binding protein	V$POU3F3.01	POU class 3 homeobox 3 (POU3F3), OTF8	67	81	74	(+)
V$HOXF	Paralog hox genes 1-8 from the four hox clusters A, B, C, D	V$HOXC8.01	Homeobox C8 / Hox-3alpha	67	85	76	(-)
V$BRN5	Brn-5 POU domain factors	V$BRN5.01	Brn-5, POU-VI protein class (also known as emb and CNS-1)	67	89	78	(+)
V$LHXF	Lim homeodomain factors	V$LHX3.01	Homeodomain binding site in LIM/Homeodomain factor LHX3	67	89	78	(-)
V$BRNF	Brn POU domain factors	V$BRN3.02	Brn-3, POU-IV protein class	68	86	77	(+)
V$HBOX	Homeobox transcription factors	V$VAX2.01	Ventral anterior homeobox 2	68	86	77	(-)
V$HOMF	Homeodomain transcription factors	V$MSX1.01	Muscle-segment homeobox 1, msh homeobox 1	68	86	77	(+)
V$HOMF	Homeodomain transcription factors	V$BARX2.01	Barx2, homeobox transcription factor that preferentially binds to paired TAAT motifs	69	87	78	(-)
V$HBOX	Homeobox transcription factors	V$VAX2.01	Ventral anterior homeobox 2	69	87	78	(+)
V$NKX1	NK1 homeobox transcription factors	V$NKX12.01	NK1 homeobox 2, Sax1-like	69	85	77	(-)
V$OCT1	Octamer binding protein	V$OCT1.03	Octamer-binding transcription factor-1, POU class 2 homeobox 1 (POU2F1)	69	83	76	(+)
V$BRNF	Brn POU domain factors	V$BRN3.03	POU class 4 homeobox 3 (POU4F3), BRN3C	69	87	78	(-)
V$CART	Cart-1 (cartilage homeoprotein 1)	V$S8.01	Binding site for S8 type homeodomains	70	90	80	(+)
V$LHXF	Lim homeodomain factors	V$LHX3.01	Homeodomain binding site in LIM/Homeodomain factor LHX3	70	92	81	(+)
V$NKX6	NK6 homeobox transcription factors	V$NKX63.01	NK6 homeobox 3	70	84	77	(+)
V$PAXH	PAX homeodomain binding sites	V$PAX6_HD.01	Paired box 6, homeodomain binding site	70	84	77	(-)
V$HOXF	Paralog hox genes 1-8 from the four hox clusters A, B, C, D	V$HOXC8.01	Homeobox C8 / Hox-3alpha	70	88	79	(+)
V$NKXH	NKX homeodomain factors	V$NKX25.02	Homeo domain factor Nkx-2.5/Csx, tinman homolog low affinity sites	70	88	79	(+)
V$ABDB	Abdominal-B type homeodomain transcription factors	V$HOXC9.01	Homeobox C9 / Hox-3beta	70	86	78	(+)

V$DLXF	Distal-less homeodomain transcription factors	V$DLX2.01	Distal-less homeobox 2	71	89	80	(-)
V$PAXH	PAX homeodomain binding sites	V$PAX4.02	Paired box 4, homeodomain binding site	71	85	78	(+)
V$HOXF	Paralog hox genes 1-8 from the four hox clusters A, B, C, D	V$HOXB8.01	Homeobox B8 / Hox-2delta	71	89	80	(-)
V$PDX1	Pancreatic and intestinal homeodomain transcription factor	V$IPF1.01	Insulin promoter factor 1, pancreatic and duodenal homeobox 1 (Pdx1)	71	89	80	(-)
V$LHXF	Lim homeodomain factors	V$LMX1B.01	LIM-homeodomain transcription factor	71	93	82	(-)
V$NKX6	NK6 homeobox transcription factors	V$NKX61.01	NK6 homeobox 1	71	85	78	(-)
V$HOXC	HOX - PBX complexes	V$HOXA9.01	Member of the vertebrate HOX - cluster of homeobox factors	72	88	80	(-)
V$OCT1	Octamer binding protein	V$OCT1.03	Octamer-binding transcription factor-1, POU class 2 homeobox 1 (POU2F1)	72	86	79	(-)
V$PIT1	GHF-1 pituitary specific pou domain transcription factor	V$PIT1.02	POU domain, class 1, transcription factor 1 (POU1F1) / Pituitary transcription factor-1	72	86	79	(-)
V$ARID	AT rich interactive domain factor	V$BRIGHT.01	Bright, B cell regulator of IgH transcription	72	92	82	(+)
V$BRNF	Brn POU domain factors	V$BRN3.03	POU class 4 homeobox 3 (POU4F3), BRN3C	72	90	81	(+)
V$LEFF	LEF1/TCF	V$LEF1.04	TCF/LEF-1 (secondary DNA binding preference)	72	88	80	(+)
V$ABDB	Abdominal-B type homeodomain transcription factors	V$HOXC9.01	Homeobox C9 / Hox-3beta	73	89	81	(-)
V$ATBF	AT-binding transcription factor	V$ATBF1.01	AT-binding transcription factor 1	73	89	81	(-)
V$PIT1	GHF-1 pituitary specific pou domain transcription factor	V$PIT1.02	POU domain, class 1, transcription factor 1 (POU1F1) / Pituitary transcription factor-1	73	87	80	(+)
V$NKX6	NK6 homeobox transcription factors	V$NKX61.01	NK6 homeobox 1	74	88	81	(+)
V$BCDF	Bicoid-like homeodomain transcription factors	V$CRX.01	Cone-rod homeobox-containing transcription factor / otx-like homeobox gene	74	90	82	(+)
V$HOXF	Paralog hox genes 1-8 from the four hox clusters A, B, C, D	V$HOXC8.01	Homeobox C8 / Hox-3alpha	74	92	83	(+)
V$HOXF	Paralog hox genes 1-8 from the four hox clusters A, B, C, D	V$HOX1-3.01	Hox-1.3, vertebrate homeobox protein	81	99	90	(-)
V$BRNF	Brn POU domain factors	V$BRN3.03	POU class 4 homeobox 3 (POU4F3), BRN3C	83	101	92	(-)

V$HOMF	Homeodomain transcription factors	V$BARX2.01	Barx2, homeobox transcription factor that preferentially binds to paired TAAT motifs	83	101	92	(-)
V$HOXF	Paralog hox genes 1-8 from the four hox clusters A, B, C, D	V$HOXB8.01	Homeobox B8 / Hox-2delta	85	103	94	(-)
V$HBOX	Homeobox transcription factors	V$GSH1.01	Homeobox transcription factor Gsh-1	86	104	95	(-)
V$HOMF	Homeodomain transcription factors	V$HMX1.01	H6 family homeobox 1 / NKX5-3	86	104	95	(+)
V$ABDB	Abdominal-B type homeodomain transcription factors	V$HOXC9.01	Homeobox C9 / Hox-3beta	87	103	95	(-)
V$NKX6	NK6 homeobox transcription factors	V$NKX61.02	NK6 homeobox 1	88	102	95	(+)
V$CART	Cart-1 (cartilage homeoprotein 1)	V$PHOX2.01	Phox2a (ARIX) and Phox2b	88	108	98	(+)
V$BCDF	Bicoid-like homeodomain transcription factors	V$CRX.01	Cone-rod homeobox-containing transcription factor / otx-like homeobox gene	88	104	96	(+)
O$TF2D	General transcription factor IID, GTF2D	O$INR_DPE.01	Initiator (INR) and downstream promoter element (DPE) with strictly maintained spacing	92	130	111	(-)
V$MAZF	Myc associated zinc fingers	V$MAZ.01	Myc associated zinc finger protein (MAZ)	94	106	100	(-)
V$ZF01	C2H2 zinc finger transcription factors 1	V$ZBRK1.01	Transcription factor with 8 central zinc fingers and an N-terminal KRAB domain	100	124	112	(-)
V$FKHD	Fork head domain factors	V$FHXB.01	Fork head homologous X binds DNA with a dual sequence specificity (FHXA and FHXB)	100	116	108	(+)
V$SORY	SOX/SRY-sex/testis determinig and related HMG box factors	V$SOX5.01	Sox-5	101	125	113	(+)
V$STAT	Signal transducer and activator of transcription	V$STAT6.01	STAT6: signal transducer and activator of transcription 6	117	135	126	(-)
V$PLAG	Pleomorphic adenoma gene	V$PLAG1.01	Pleomorphic adenoma gene (PLAG) 1, a developmentally regulated C2H2 zinc finger protein	128	150	139	(-)
V$AP4R	AP4 and related proteins	V$AP4.02	Activator protein 4	129	145	137	(-)
V$MYOD	Myoblast determining factors	V$MYOGENIN.02	Myogenic bHLH protein myogenin (myf4)	129	145	137	(+)
V$AP4R	AP4 and related proteins	V$AP4.01	Activator protein 4	130	146	138	(+)
V$MYOD	Myoblast determining factors	V$MYOGENIN.02	Myogenic bHLH protein myogenin (myf4)	130	146	138	(-)
V$NEUR	NeuroD, Beta2, HLH domain	V$ATOH1.01	Atonal homolog 1, HATH1, MATH-1	131	143	137	(+)

V$HOXF	Paralog hox genes 1-8 from the four hox clusters A, B, C, D	V$NANOG.01	Homeobox transcription factor Nanog	144	162	153	(+)
V$SORY	SOX/SRY-sex/testis determinig and related HMG box factors	V$HBP1.02	HMG box-containing protein 1	144	168	156	(+)
V$NFAT	Nuclear factor of activated T-cells	V$NFAT.01	Nuclear factor of activated T-cells	153	171	162	(+)
V$FKHD	Fork head domain factors	V$FHXB.01	Fork head homologous X binds DNA with a dual sequence specificity (FHXA and FHXB)	156	172	164	(+)
V$BRNF	Brn POU domain factors	V$TST1.01	POU-factor Tst-1/Oct-6	156	174	165	(+)
V$EVI1	EVI1-myleoid transforming protein	V$MEL1.01	MEL1 (MDS1/EVI1-like gene 1) DNA-binding domain 1	159	175	167	(+)
V$HOXC	HOX - PBX complexes	V$HOXC9.02	Member of the vertebrate HOX - cluster of homeobox factors	161	177	169	(+)
V$RORA	v-ERB and RAR-related orphan receptor alpha	V$RORA2.01	RAR-related orphan receptor alpha2	163	185	174	(+)
V$PAX5	PAX-2/5/8 binding sites	V$PAX2.02	Paired box protein 2	163	191	177	(-)
V$HICF	Krueppel-like C2H2 zinc finger factors hypermethylated in cancer	V$HIC1.02	Hypermethylated in cancer 1 (secondary DNA binding preference)	167	179	173	(-)
V$SIXF	Sine oculis (SIX) homeodomain factors	V$SIX6.01	Sine oculis homeobox homolog 6, optic homeobox 2 (OPTX2)	168	182	175	(+)
V$HEAT	Heat shock factors	V$HSF1.04	Heat shock factor 1	176	200	188	(+)
V$WHNF	Winged helix binding sites	V$WHN.01	Winged helix protein, involved in hair keratinization and thymus epithelium differentiation	177	187	182	(-)
V$HNFP	Histone nuclear factor P	V$MIZF.01	MBD2 (methyl-CpG-binding protein)-interacting zinc finger protein, histone nuclear factor P (HiNF-P)	177	189	183	(-)
V$HEAT	Heat shock factors	V$HSF2.02	Heat shock factor 2	185	209	197	(-)
V$PLAG	Pleomorphic adenoma gene	V$PLAG1.01	Pleomorphic adenoma gene (PLAG) 1, a developmentally regulated C2H2 zinc finger protein	187	209	198	(+)
V$CEBP	Ccaat/Enhancer Binding Protein	V$CEBP.02	CCAAT/enhancer binding protein	190	204	197	(+)
V$MOKF	Mouse Krueppel like factor	V$MOK2.01	Ribonucleoprotein associated zinc finger protein MOK-2 (mouse)	196	216	206	(-)
V$ZF02	C2H2 zinc finger transcription factors 2	V$ZBP89.01	Zinc finger transcription factor ZBP-89	197	219	208	(-)
V$INSM	Insulinoma associated factors	V$INSM1.01	Zinc finger protein insulinoma-associated 1 (IA-1) functions as a transcriptional repressor	197	209	203	(+)
V$NR2F	Nuclear receptor subfamily 2 factors	V$HPF1.01	HepG2-specific P450 2C factor-1, DR1 sites	199	223	211	(+)

V$PLAG	Pleomorphic adenoma gene	V$PLAG1.02	Pleomorphic adenoma gene 1	200	222	211	(+)
V$ZF02	C2H2 zinc finger transcription factors 2	V$ZF9.01	Core promoter-binding protein (CPBP) with 3 Krueppel-type zinc fingers	204	226	215	(+)
V$ZF02	C2H2 zinc finger transcription factors 2	V$ZBTB7.01	Zinc finger and BTB domain containing 7, Proto-oncogene FBI-1, Pokemon	207	229	218	(+)
V$MOKF	Mouse Krueppel like factor	V$MOK2.02	Ribonucleoprotein associated zinc finger protein MOK-2 (human)	207	227	217	(-)
V$GLIF	GLI zinc finger family	V$GLIS3.01	GLIS family zinc finger 3, Gli-similar 3	211	225	218	(+)
V$SP1F	GC-Box factors SP1/GC	V$GC.01	GC box elements	211	227	219	(-)
V$MAZF	Myc associated zinc fingers	V$MAZR.01	MYC-associated zinc finger protein related transcription factor	212	224	218	(-)
V$RREB	Ras-responsive element binding protein	V$RREB1.01	Ras-responsive element binding protein 1	219	233	226	(+)
V$BARB	Barbiturate-inducible element box from pro+eukaryotic genes	V$BARBIE.01	Barbiturate-inducible element	240	254	247	(+)
V$BRN5	Brn-5 POU domain factors	V$BRN5.03	Brn-5, POU-VI protein class (also known as emb and CNS-1)	242	264	253	(-)
V$SORY	SOX/SRY-sex/testis determinig and related HMG box factors	V$HBP1.01	HMG box-containing protein 1	242	266	254	(-)
V$MTF1	Metal induced transcription factor	V$MTF-1.02	Metal-regulatory transcription factor 1	245	259	252	(-)
V$HESF	Vertebrate homologues of enhancer of split complex	V$HES1.01	Drosophila hairy and enhancer of split homologue 1 (HES-1)	245	259	252	(+)
V$FAST	FAST-1 SMAD interacting proteins	V$FAST1.02	Forkhead box H1 (Foxh1)	246	262	254	(+)
V$HOXF	Paralog hox genes 1-8 from the four hox clusters A, B, C, D	V$NANOG.01	Homeobox transcription factor Nanog	247	265	256	(-)
V$STEM	Motif composed of binding sites for pluripotency or stem cell factors	V$OCT3_4.02	POU domain, class 5, transcription factor 1	247	265	256	(+)
V$CART	Cart-1 (cartilage homeoprotein 1)	V$PHOX2.01	Phox2a (ARIX) and Phox2b	248	268	258	(-)
V$LHXF	Lim homeodomain factors	V$LHX6.01	LIM homeobox 6	249	271	260	(+)
V$HOXF	Paralog hox genes 1-8 from the four hox clusters A, B, C, D	V$HOXA3.01	Homeobox A3	250	268	259	(-)
V$LHXF	Lim homeodomain factors	V$LHX6.01	LIM homeobox 6	250	272	261	(-)
V$HBOX	Homeobox transcription factors	V$EVX1.01	Even-skipped homeobox 1	251	269	260	(-)
V$BRNF	Brn POU domain factors	V$BRN3.03	POU class 4 homeobox 3 (POU4F3), BRN3C	252	270	261	(-)

V$HBOX	Homeobox transcription factors	V$EVX1.01	Even-skipped homeobox 1	252	270	261	(+)
V$HOXF	Paralog hox genes 1-8 from the four hox clusters A, B, C, D	V$HOXB3.01	Homeobox B3 / Hox 2-gamma	253	271	262	(+)
V$CART	Cart-1 (cartilage homeoprotein 1)	V$RHOX6.01	Reproductive homeobox 6, placenta specific homeobox 1	253	273	263	(+)
V$NKX6	NK6 homeobox transcription factors	V$NKX63.01	NK6 homeobox 3	254	268	261	(-)
V$PDX1	Pancreatic and intestinal homeodomain transcription factor	V$PDX1.01	Pdx1 (IDX1/IPF1) pancreatic and intestinal homeodomain TF	254	272	263	(-)
V$HBOX	Homeobox transcription factors	V$GSH1.01	Homeobox transcription factor Gsh-1	255	273	264	(-)
V$TALE	TALE homeodomain class recognizing TG motifs	V$TGIF.01	TG-interacting factor belonging to TALE class of homeodomain factors	264	280	272	(-)
V$NR2F	Nuclear receptor subfamily 2 factors	V$HNF4.01	Hepatic nuclear factor 4, DR1 sites	277	301	289	(-)
V$DEAF	Homolog to deformed epidermal autoregulatory factor-1 from D. melanogaster	V$NUDR.01	NUDR (nuclear DEAF-1 related transcriptional regulator protein)	280	298	289	(+)
V$RXRF	RXR heterodimer binding sites	V$VDR_RXR.03	Bipartite binding site of VDR/RXR heterodimers, DR1 sites	286	310	298	(-)
V$CLOX	CLOX and CLOX homology (CDP) factors	V$CDP.02	Transcriptional repressor CDP	288	306	297	(+)
V$ABDB	Abdominal-B type homeodomain transcription factors	V$HOXC13.01	Homeodomain transcription factor HOXC13	288	304	296	(+)
V$CAAT	CCAAT binding factors	V$NFY.04	Nuclear factor Y (Y-box binding factor)	289	303	296	(+)
V$CDXF	Vertebrate caudal related homeodomain protein	V$CDX2.03	Caudal type homeobox transcription factor 2	289	307	298	(-)
V$FAST	FAST-1 SMAD interacting proteins	V$FAST1.01	FAST-1 SMAD interacting protein	291	307	299	(-)
V$AP1R	MAF and AP1 related factors	V$MAFK.01	V-maf musculoaponeurotic fibrosarcoma oncogene homolog K (half site)	295	315	305	(+)
V$FXRE	Farnesoid X - activated receptor response elements	V$FXRE.01	Farnesoid X - activated receptor (RXR/FXR dimer), IR1 sites	302	314	308	(-)
V$ARID	AT rich interactive domain factor	V$BRIGHT.01	Bright, B cell regulator of IgH transcription	306	326	316	(-)
V$BRNF	Brn POU domain factors	V$BRN4.01	POU domain transcription factor brain 4	307	325	316	(+)
V$HOMF	Homeodomain transcription factors	V$HHEX.01	Hematopoietically expressed homeobox, proline-rich homeodomain protein	307	325	316	(+)
V$CART	Cart-1 (cartilage homeoprotein 1)	V$ISX.01	Intestine-specific homeobox	308	328	318	(-)
V$BRNF	Brn POU domain factors	V$TST1.01	POU-factor Tst-1/Oct-6	308	326	317	(-)

V$LHXF	Lim homeodomain factors	V$LHX8.01	LIM homeobox 8	309	331	320	(+)
V$DLXF	Distal-less homeodomain transcription factors	V$DLX3.01	Distal-less 3 homeodomain transcription factor	309	327	318	(+)
V$HOXF	Paralog hox genes 1-8 from the four hox clusters A, B, C, D	V$HOXA3.02	Homeobox A3	310	328	319	(-)
V$LHXF	Lim homeodomain factors	V$ISL2.01	ISL LIM homeobox 2	310	332	321	(-)
V$CLOX	CLOX and CLOX homology (CDP) factors	V$CDP.02	Transcriptional repressor CDP	311	329	320	(-)
V$HBOX	Homeobox transcription factors	V$VAX2.01	Ventral anterior homeobox 2	311	329	320	(-)
V$HOMF	Homeodomain transcription factors	V$NOBOX.01	Homeobox containing germ cell-specific transcription factor NOBOX	311	329	320	(+)
V$HBOX	Homeobox transcription factors	V$GSH2.01	Homeodomain transcription factor Gsh-2	312	330	321	(+)
V$HOMF	Homeodomain transcription factors	V$NOBOX.02	NOBOX oogenesis homeobox	312	330	321	(-)
V$NKX6	NK6 homeobox transcription factors	V$NKX61.01	NK6 homeobox 1	313	327	320	(+)
V$BCDF	Bicoid-like homeodomain transcription factors	V$PCE1.01	Photoreceptor conserved element 1	313	329	321	(+)
V$CART	Cart-1 (cartilage homeoprotein 1)	V$S8.01	Binding site for S8 type homeodomains	313	333	323	(+)
V$HOXF	Paralog hox genes 1-8 from the four hox clusters A, B, C, D	V$HOXA3.01	Homeobox A3	313	331	322	(+)
V$NKXH	NKX homeodomain factors	V$NKX25.02	Homeo domain factor Nkx-2.5/Csx, tinman homolog low affinity sites	313	331	322	(+)
V$CAAT	CCAAT binding factors	V$CAAT.01	Cellular and viral CCAAT box	314	328	321	(-)
V$DLXF	Distal-less homeodomain transcription factors	V$DLX5.01	Distal-less homeobox 5	314	332	323	(-)
O$VTBP	Vertebrate TATA binding protein factor	O$ATATA.01	Avian C-type LTR TATA box	319	335	327	(-)
V$GREF	Glucocorticoid responsive and related elements	V$ARE.03	Androgene receptor binding site, IR3 sites	327	345	336	(-)
V$SP1F	GC-Box factors SP1/GC	V$SP1.03	Stimulating protein 1, ubiquitous zinc finger transcription factor	330	346	338	(+)
V$E2FF	E2F-myc activator/cell cycle regulator	V$E2F3.01	E2F transcription factor 3	332	348	340	(+)
V$PAX5	PAX-2/5/8 binding sites	V$PAX5.01	B-cell-specific activator protein	339	367	353	(+)
V$SP1F	GC-Box factors SP1/GC	V$GC.01	GC box elements	347	363	355	(+)
V$ZF02	C2H2 zinc finger transcription factors 2	V$ZKSCAN3.01	Zinc finger with KRAB and SCAN domains 3	348	370	359	(-)
V$MAZF	Myc associated zinc fingers	V$MAZR.01	MYC-associated zinc finger protein related transcription factor	350	362	356	(+)

V$KLFS	Krueppel like transcription factors	V$KLF6.01	Kruppel-like factor 6	350	366	358	(+)
V$NRSF	Neuron-restrictive silencer factor	V$NRSF.03	Neuron-restrictive silencer factor (17 bp spacer between half sites)	357	387	372	(-)
V$AP2F	Activator protein 2	V$TCFAP2C.02	Transcription factor AP-2, gamma	363	377	370	(-)
V$RU49	Zinc finger transcription factor RU49, zinc finger proliferation 1 - Zipro1	V$RU49.01	Zinc finger transcription factor RU49 (zinc finger proliferation 1 - Zipro 1). RU49 exhibits a strong preference for binding to tandem repeats of the minimal RU49 consensus binding site.	377	383	380	(-)
